# Evolution of snow algae, from cosmopolitans to endemics, revealed by DNA analysis of ancient ice

**DOI:** 10.1038/s41396-023-01359-3

**Published:** 2023-01-17

**Authors:** Takahiro Segawa, Takahiro Yonezawa, Ryo Matsuzaki, Hiroshi Mori, Ayumi Akiyoshi, Francisco Navarro, Koji Fujita, Vladimir B. Aizen, Zhongqin Li, Shuhei Mano, Nozomu Takeuchi

**Affiliations:** 1 Center for Life Science Research, University of Yamanashi, Yamanashi, Japan; 2 Department of Animal Science, Faculty of Agriculture, Tokyo University of Agriculture, Kanagawa, Japan; 3 Faculty of Life and Environmental Sciences, University of Tsukuba, Ibaraki, Japan; 4 Biodiversity Division, National Institute for Environmental Studies, Ibaraki, Japan; 5 Advanced Genomics Center, National Institute of Genetics, Shizuoka, Japan; 6 National Institute of Polar Research, Tokyo, Japan; 7 Departamento de Matemática Aplicada a las Tecnologías de la Información y las Comunicaciones, ETSI de Telecomunicación, Universidad Politécnica de Madrid, Madrid, Spain; 8 Graduate School of Environmental Studies, Nagoya University, Aichi, Japan; 9 Department of Earth and Space Science, University of Idaho, Moscow, Idaho USA; 10 Laboratory of Cryospheric Sciences, Northwest Institute of Eco-Environment and National Resources/Tianshan Glaciological Station, Chinese Academy of Sciences, Gansu, China; 11 The Institute of Statistical Mathematics, Tokyo, Japan; 12 Department of Earth Sciences, Graduate School of Science, Chiba University, Chiba, Japan

**Keywords:** Phylogenetics, Biogeography, Molecular evolution

## Abstract

Recent studies of microbial biogeography have revealed the global distribution of cosmopolitans and dispersal of regional endemics, but little is known about how these processes are affected by microbial evolution. Here, we compared DNA sequences from snow/glacier algae found in an 8000-year-old ice from a glacier in central Asia with those from modern snow samples collected at 34 snow samples from globally distributed sites at the poles and mid-latitudes, to determine the evolutionary relationship between cosmopolitan and endemic phylotypes of snow algae. We further applied a coalescent theory–based demographic model to the DNA sequences. We found that the genus *Raphidonema* (Trebouxiophyceae) was distributed over both poles and mid-latitude regions and was detected in different ice core layers, corresponding to distinct time periods. Our results indicate that the modern cosmopolitan phylotypes belonging to *Raphidonema* were persistently present long before the last glacial period. Furthermore, endemic phylotypes originated from ancestral cosmopolitan phylotypes, suggesting that modern regional diversity of snow algae in the cryosphere is a product of microevolution. These findings suggest that the cosmopolitans dispersed across the world and then derived new localized endemics, which thus improves our understanding of microbial community formation by microevolution in natural environments.

## Introduction

Global microbial colonization, which gives rise to cosmopolitan species, has been a topic of great interest since 1934, when Baas Becking hypothesized that “Everything is everywhere, but the environment selects” [1]. However, few details are known about the spatial genetic structures within microbial cosmopolitan species as well as the processes by which they have formed. Advances in genome sequencing technology have revealed that most of the observed spatial distribution of microorganisms has been endemic, being found only in localized regions, based on DNA sequencing at single-nucleotide (nt) resolution [2–5]. Moreover, a recent study of photosynthetic algae that grow on the snow surface in polar regions demonstrated that these snow algae consist of limited numbers of cosmopolitan phylotypes and very diverse endemic phylotypes [6]. Current global diversification and regional endemism of microorganisms have been formed by genetic isolation and adaptation [7–10]. Therefore, to understand how microbes migrate globally and adapt locally, it is necessary to study the evolutionary history of cosmopolitan and endemic phylotypes.

Our knowledge on the time scale of development of microbial population structures, and how they have been preserved is very limited. Because ice-core samples from glaciers provide a time series of past genome information [11], analysis of the DNA information in ice core samples offers new insights into the micro-evolutionary process that shapes the global-scale biogeographic structure. In addition, because the snow microbiota is genetically tractable, and the polar and mid-latitude/high-mountain cryosphere regions are physically isolated, this microbiota is most suitable for understanding the microevolution of communities driven by migration-isolation and subsequent local adaptation [12–15]. Therefore, microorganisms in snow and ice environments are the most appropriate organisms for elucidating the history of biogeographical distribution and microevolution. Although microbial analyses of ice cores have been conducted using conserved gene regions such as 16S or 18S rRNA genes [16–22], no analysis has focused on the microevolution between cosmopolitans and endemic distribution.

To overcome this shortcoming, we here analyze ancient DNA information derived from an ice-core that dates continuously back to 8000 years before the present, and analyze a comprehensive global modern dataset of snow algae collected from both poles and mid-latitudes (Tables S1–S2). The results clarify the time scale on which cosmopolitan species were established and maintained.

## Materials and methods

### Ice-core samples

**Fig. 1:**
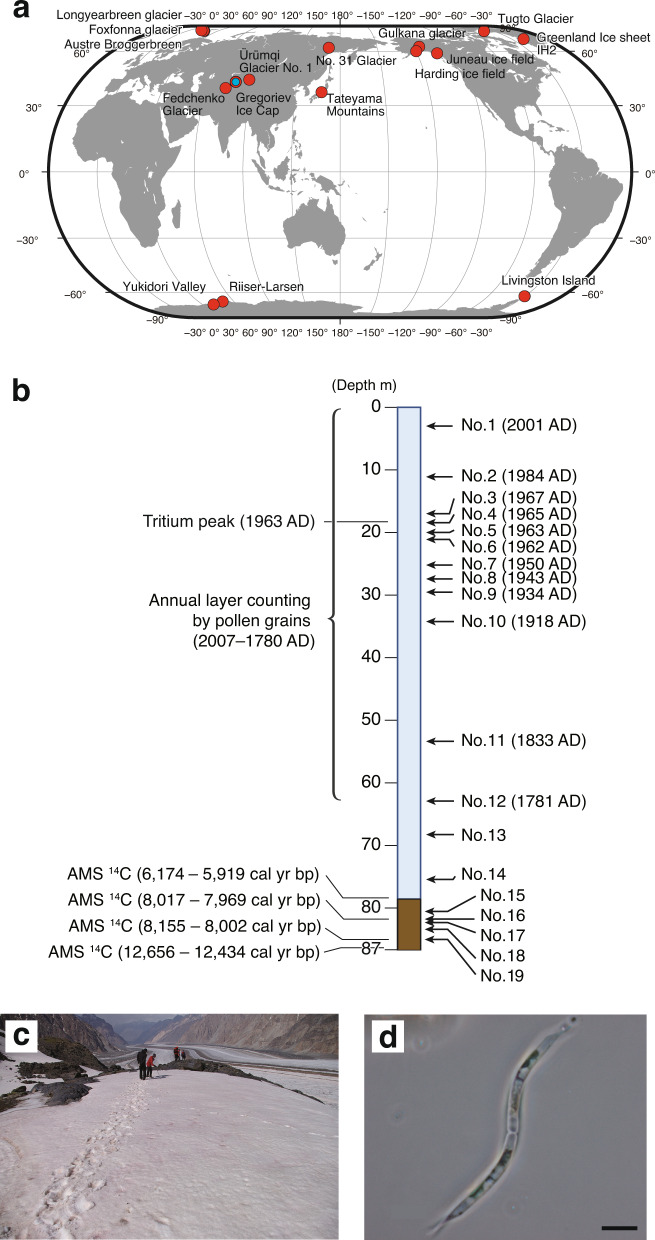
Collection sites of red snow and ice core samples, ice core information, and photographs of a study site and of a snow alga. **a** Locations of the Grigoriev Ice Cap in the Tien Shan Mountains in the Kyrgyz Republic, where the ice core was drilled (blue circle), and of snow sampling sites (red circles, Tables S1–S2). **b** Schematic drawing of the ice core, with indication of depth from the surface, the dating age of the core, and the positions of the samples analyzed. The depth (in meters) and dating of the dust layer sections are also shown. **c** Red snow on a study site at mid-laditudes (Fedchenko Glacier, Pamir). **d** Microscopic view of *Raphidonema* sp. (collected on Gulkana Glacier, Alaska). Scale bar, 10 μm.

The ice core was drilled at the top of the Grigoriev Ice Cap in the Tien Shan Mountains in Kyrgyz Republic in August, 2007 (Fig. 1). The ice cap summit is a flat snowfield at elevation 4563 m a.s.l. [23]. The 86.87 m length ice core, made up of 19 pieces, spanned the distance from the surface to the bedrock of the ice cap. The ice core was transported in a frozen state to the ice-core laboratory at the Research Institute for Humanity and Nature, Japan. The bottom age was dated as 12,500 calendar years before present (1950 AD) based on radiocarbon dating of particulate organic matter [24].

The 19 ice-core samples were melted using a device developed by our group that enabled us to obtain water from only the inner portion of the core; the 6-mm-thick outer cylindrical layer remained intact [25]. Complete separation of the inner and outer portions of the ice core is required to avoid contamination by modern microorganisms that can adhere to a core during drilling and storage. The resultant water samples (10–20 ml) containing visible dust particles were filtered through a sterilized 0.22 µm membrane filter (Nalgene Analytical Filter Unit, Thermo Scientific, USA) to collect microorganisms.

To determine whether any contamination had occurred during handling or due to ice-core cracks, a solution of ~2 ng/µl bacterial plasmid vector (pCR4-TOPO vector, Life Technologies, Carlsbad, CA, USA) was applied to the ice-core surface for 20 h before sampling of the inner portion. The vector contaminant was not amplified from the inner part of ice-core samples, but amplified only for the outer layer of ice-core samples as assessed by 45 cycles of PCR with vector-specific primers (M13-Forward and M13-Reverse primers).

### Modern snow samples

Snow samples were collected into sterile 50 ml plastic conical tubes during the melt season from 10 sites on 5 glaciers or snowpacks in No. 31 Glacier in Suntar-Khayata Mountains in Russia; Glacier No. 1 in the Eastern Tien Shan in China; the Grigoriev Ice cap at the Inner Tien Shan in Kyrgyz Republic; Fedchenko Glacier in Central Pamir in Republic of Tajikistan; and Tateyama Mountains in Japan (Table S2). Samples were kept frozen during transport to the National Institute of Polar Research (Tokyo, Japan) and then stored at –80 °C before use. Although phototrophs of glaciers include both snow and glacier algae, which dominate on snow-covered and bare ice surfaces of the glacier, respectively, in this study we simply refer to them as snow algae since most of the samples analyzed were collected in snow-covered surfaces.

### DNA extraction

Prior to genome library amplification, all work on ancient DNA in ice cores was conducted in a dedicated clean room for ancient DNA at the National Institute of Polar Research, Japan. Membrane filters containing microbial DNA were transferred into 2 ml Matrix-E tubes (Qbiogene, USA) with 600 µl of extraction buffer as described in Willerslev et al. [16]. Two sequential homogenizations were carried out with a multi-beads shocker (Yasui Kikai, Japan) at 1000 rpm for 30 s to remove microorganisms and then at 2500 rpm for 30 s to disrupt microorganisms. The homogenized samples were added with proteinase K (Roche) and incubated at 55 °C for at least 4 h under agitation. Each solution was adjusted with NaCl to 1.15 M, treated with ½ vol. of chloroform/octanol (24:1), and slowly agitated overnight at room temperature. The mixture was centrifuged at 12,000 × *g* for 2 min, and the aqueous phase was collected and transferred into a separate microtube for incubation at 2 °C for at least 1 h. Further purification steps were the same as described by Orlando et al. [26]. The supernatant was incubated with 100 μl silica beads and 40 ml of binding buffer (Qiagen PB buffer; 25 mM NaCl, 87 mM Na acetate) for 3 h at room temperature. The supernatant was discarded, and the pellet was washed twice with 1 ml of 80% ethanol before eluting the DNA with 60 μl elution buffer (Qiagen, Germany).

The modern snow samples were melted at 4 °C, and 5–10 ml of each sample were centrifuged at 5000 × *g* for 10 min to obtain a pellet. The pellets from five replicate samples collected at each site were pooled and used for DNA extraction. Genomic DNA was extracted from each pellet using a FastDNA spin kit for soil (Qbiogene) with a Multi-beads shocker (Yasui Kikai) at 2500 rpm for 30 s. All DNA extractions were conducted on a class 100 clean bench (Sanyo, Japan), and subsequent procedures were carried out on another class 100 clean bench.

### ITS2 region amplicon sequencing

The ITS2 region was amplified by PCR using primers c and b (5′-GCATCGATGAAGAACGCAGC-3′ and 5′-GGGATCCATATGCTTAAGTTCAGCGGGT-3′, respectively; [27]) with Illumina overhang adaptor sequences at their 5′ ends. Each PCR mixture (25 μl) contained 1 × KAPA HiFi HS ReadyMix (Kapa Biosystems, USA), 0.2 μM of each primer, and 2–4 μl template DNA. The PCR cycling conditions were as follows: initial annealing at 95 °C for 3 min, followed by 25 cycles (for modern samples) or 45 cycles (ice-core samples) at 95 °C for 30 s, 50 °C for 30 s, and 72 °C for 60 s, with a final extension at 72 °C for 5 min. The amplicons with Illumina overhang adapter sequences were generated in triplicate, and each amplicon was pooled before index PCR. The PCR products were labeled with two sample-specific indices each containing a sample-unique index [28] and Illumina adapter sequences at their 5′ end (Nextera XT index kit v2, Illumina). The PCR mixture (10 μl) contained 1×KAPA HiFi HS ReadyMix, 2 μl each of forward and reverse primers, and 1 μl of the recovered PCR products. PCR was performed under the following cycling conditions: 95 °C for 3 min, followed by 8 cycles of 95 °C for 30 s, 55 °C for 30 s, and 72 °C for 60 s, with final extension at 72 °C for 5 min. After agarose gel electrophoresis, PCR products were excised from the gel and purified using the NucleoSpin Gel and PCR clean-up kit (Macherey-Nagel, Germany). Tagged amplicons were mixed with PhiX control DNA at a ratio of 80:20 and used as a template for MiSeq paired-end sequencing (2 × 300 bp) using Reagent kit v3 (Illumina). To avoid index switching with MiSeq [28], two separate MiSeq runs were carried out for the ice-core and modern samples.

### Sequence quality filtering and taxonomic assignments

Adapter sequences in the reads were removed, and quality filtering was conducted using fastp version 0.20 with default parameters [29]. The forward and reverse MiSeq reads were merged for the paired-end libraries of ITS2 using USEARCH (version 10.0.240) [30] with the fastq_mergepairs parameter. Only paired reads carrying the exact index combinations were assigned to each sample’s reads and used for subsequent analyses. Reads were discarded if they (i) contained ambiguous nts, and/or (ii) mapped to the PhiX genome sequence as determined by Bowtie 2 (version 2.2.4) with default parameters [31]. The forward and reverse PCR primer sequences were removed with cutadapt (version 2.6) with default parameters [32].

The unique ITS2 sequence clusters, i.e., the non-redundant sequence clusters, were constructed by clustering high-quality reads of all samples using USEARCH (version 10.0.240) with the fastx_uniques parameter. The unique sequences of the ITS2 region were aligned using MAFFT v7.429 [33]. These alignments were carefully inspected by eye, and incomplete sequences and those that were not full length and the singleton clusters were discarded.

Taxonomic assignments of unique ITS2 sequences were conducted by a BLASTn search [34] with a top-hit *E* value of <1e^–8^, identity of >90%, and alignment length of >200 bp against (i) the UNITE fungal ITS2 sequence database [35], (ii) Viridiplantae ITS2 sequences obtained from NCBI, and (iii) unique ITS2 sequences of snow algae detected from red snow in a previous study [6]. Sequence clustering was then conducted with a 98% nt identity level based on the ITS2 sequences in each sample, using the furthest neighbor algorithm in Mothur 1.44.1 [36].

Using the unique sequences of the ITS2 region detected from each geographical region, we divided the distribution into three types: cosmopolitans, multi-regions, and endemics. Cosmopolitan types are distributed at both poles and middle latitudes (detected in Antarctica, Svalbard, Greenland, Alaska, and mid-latitude regions); multi-region types are distributed in two regions (Antarctica and a mid-latitude region, the Arctic and a mid-latitude region, or at both poles only). The endemic types are distributed in one region (either the Arctic, Antarctica, or a mid-latitude region) (Table S8). In addition, ice core samples were investigated for cosmopolitan/endemic phylotypes in the past.

We compared the number of unique sequences obtained by usearch with the amplicon sequence variants (ASVs) obtained by DADA2. All sequences were clustered into ASVs using the R package DADA2 [37] by following the procedure on the DADA2 analysis tutorial web page.

### Groups based on the secondary structure of the ITS2 region

For estimating the diversity of snow algae within the snow samples, the ITS2 region sequences were classified at the species level according to the genetic species concept based on structural differences in the ITS2 region [38]. Hereafter, these unique sequences are defined as “phylotypes” and ITS2 sequences with ≥98% nt sequence identity as operational taxonomic units (OTUs). The 5.8S-28S rRNA interaction region within the sequence of each OTU was annotated using the web interface for Hidden Markov Models–based annotation [39] with the ITS2 database [40]. Secondary structures for ITS2 were predicted using Centroidfold [41] and RNAfold WebServer [42] and were manually refined. We confirmed that the ITS2 secondary structures of the OTUs examined in this study contained four helices as well as a U-U mismatch in helix II and a YGGY motif on the 5′ side near the apex of helix III, which are common structural hallmarks of eukaryotic ITS2 sequences [43, 44]. The ITS2 sequences of the OTUs were then compared with published sequences using BLASTn in the NCBI non-redundant nt database. Based on the BLASTn results, the OTUs were classified into five chlorophycean and six trebouxiophycean groups (Fig. S1 and Tables S3–S4). Within each group, species boundaries among the OTUs were estimated based on the compensatory base change near the apex of helix III encompassing the YGGY motif in the ITS2 secondary structure (the most conserved region of the ITS2 secondary structure of eukaryotes) [45]; the compensatory base change correlates with the separation of biological species [38].

Chimeric clusters were primarily removed by BLASTn to the NCBI nt database. After grouping the sequences based on the secondary structures, the possible chimeric sequences were checked manually and deleted. The unique sequences and OTUs of the algal ITS2 sequence clusters were identified from the taxonomic assignment results, and the remaining unique sequences and OTUs of ITS2 that were not assigned to algae were discarded.

### Phylotype network of ITS2 sequences in the *Raphidonema* group

The unique ITS2 sequences of the *Raphidonema* group in the mid-latitudes and the ice core from this study, together with the previous ITS2 sequences of snow algae in Antarctica and the Arctic [6] and those from the NCBI nt database (BLASTn searches were performed to identify the closest sequences in the NCBI nt database, i.e., those with >98.0% similarity) were aligned using the program MAFFT v7.429 [33]. These alignments were carefully checked by eye, and all ambiguous sites and sequences were manually deleted. Sequence clustering was then conducted with ≥98% nt sequence identity using the furthest neighbor algorithm in Mothur 1.44.1 [36]. The maximum-likelihood (ML) tree was inferred using IQ-tree ver. 1.6.12 [46] with the K3P + G4 model, and 1000 replications were carried out for standard bootstrap analysis.

For analyzing the evolutionary approaches of the *Raphidonema* group, the OTUs that were defined with ≥98% nt sequence identity among sequences were subdivided into five subgroups (Groups A–E) based on a phylogenetic analysis. Using the unique sequences of each subgroup, we performed the following molecular evolutionary analyses. Phylotype networks of unique sequences were then constructed within the *Raphidonema* group with the median-joining (MJ) method [47] using NETWORK 4.6.11 (http://www.fluxus-engineering.com/sharenet.htm). Because the number of unique sequences was too large for computational and visual network analysis, 200 unique sequences were used for network analysis, including the top 100 unique sequences with the largest number of reads plus 100 randomly picked unique sequences from the 101st and later unique sequences. We additionally generated multiple sets of random 101st–200th sequences by random selection and confirmed that all results were consistent.

Pairwise differences among the four regions (Antarctica, Arctic, mid-latitudes, and the ice core), and ice cores between the newer (1800–2001 AD) and the older (6000–8000 years before present) layers with respect to unique sequence cluster compositions were statistically analyzed with permutational multivariate analysis of variance (PERMANOVA) with R (version 3.6.1) and the VEGAN package.

### Inference of demography based on the coalescent model

Let us consider a simple model of demography: a population expanded *t*_0_ generations ago, and the sizes before and after the expansion were *N*_0_ and *N*_1_, respectively. The time *s* > 0 in terms of generations is measured backward from the present. The population size is *N*_0_ for *s* ≥ *t*_0_ and *N*_1_ for *s* < *t*_0_. Rogers and Harpending [48] derived a partial differential equation that is satisfied by an approximation of the probability that the number of mismatched sites between two randomly chosen sequences is *k* in generation *s* for sufficiently large *k*. However, their approximation is not justified for small *k*. Moreover, their estimation fits the solution curve to the histogram, for which statistical assessment of the estimation is difficult.

Let *K* be the number of mismatched sites between two randomly chosen sequences. The probability density of the coalescence time for two sequences is: $f\left( s \right) = e^{ - t_0/(2N_1)}{\textstyle{1 \over {2N_0}}}e^{ - (s - t_0)/(2N_0)}$ for *s* ≥ *t*_0_ and ${\textstyle{1 \over {2N_1}}}e^{ - s/(2N_1)}$ for *s* < *t*_0_. Given *s*, we assume that the number of mutations along the lineages to the most recent common ancestor of the two sequences follows a Poisson distribution of intensity 2*us*, where *u* is the mutation rate of the sequence per generation. Then, the probability mass function of *K* is given as

$$P\left( {K = k} \right) 	= \mathop {\int}\limits_0^\infty {f\left( s \right)\frac{{e^{ - 2us}}}{{k!}}\left( {2us} \right)^kds} \\ 	 = \left( {\frac{{\theta _1}}{{1 + \theta _1}}} \right)^k\frac{1}{{1 + \theta _1}}\frac{1}{{k!}}\gamma \left( {k + 1,\tau \left( {1 + \frac{1}{{\theta _1}}} \right)} \right) \\ 	 +\! \left( {\frac{{\theta _0}}{{1 + \theta _0}}} \right)^k\frac{1}{{1 + \theta _0}}\frac{1}{{k!}} {\Gamma}\left( {k + 1,\tau \left( {1 + \frac{1}{{\theta _0}}} \right)} \right)e^{ - \tau \left( {\frac{1}{{\theta _1}} - \frac{1}{{\theta _0}}} \right)},\\ $$

where *γ* and Γ are incomplete gamma functions defined as Γ $\left( {c,x} \right) = {\int}_0^x {e^{ - z}z^{c - 1}dz = 1 - \gamma \left( {c,x} \right)}$, $\tau = 2ut_0$, $\theta _0 = 2N_0u$, and $\theta _1 = 2N_1u$. The expectation $E\left( K \right) = {\int}_0^\infty {f\left( s \right)2usds}$ = $\theta _1 + (\theta _0 - \theta _1)e^{ - \tau /\theta _1}$ reproduces Tajima’s result [49].

The probability mass function of *K* may be used as the likelihood; let it be denoted as $L\left( {k;\theta _0,\theta _1,\tau } \right)$. We conducted maximum likelihood estimation based on a composite log-likelihood $\mathop {\sum}\nolimits_{i = 1}^{\left\lfloor {n/2} \right\rfloor } {\log L(k_{2i - 1,2i};\theta _0,\theta _1,\tau )}$, where $k_{2i - 1,2i}$ is the number of mismatched sites between the (2*i*−1)-th and 2*i*-th phylotypes in randomly ordered phylotypes. The reason why we did not exhaust all pairs is twofold: to avoid computational burden and to avoid dependence between pairs. The sampling variance-covariance matrix was estimated as the inverse of the observed Fisher information matrix. Note that the composite log-likelihood is still not the true likelihood because any pair of sequences taken from a population may share edges in a genealogy. The correlation among genealogies may underestimate the variances. See Larribe and Fearnhead [50] for further discussion.

The maximum likelihood estimates of τ, *θ*_0_ and *θ*_1_ are shown in Table S13 with standard deviation values. For the cosmopolitans, the standard deviations of *θ*_0_ and *θ*_1_ were not available because the likelihood was constant for *θ*_0_∈[0.108,0.010]. If the standard deviation was estimated to be a tiny value, it was omitted. The maximum likelihood estimate provides estimates of $\tau = 2ut_0$,$\theta _0 = 2N_0u$, and $\theta _1 = 2N_1u$, but we were interested in estimates of *t*_0_, *N*_0_, and *N*_1_. To derive estimates of *N*_0_, *N*_1_, and *t*_0_ in years, the mutation rate and the generation time are needed. Ness et al. [51] estimated the single-base mutation rate of the unicellular green alga *Chlamydomonas reinhardtii* to be 2.08 × 10^−10^ (95% CI: 1.09 × 10^–10^ to 3.74 × 10^–10^) per site per generation based on a mutation accumulation experiment followed by whole-genome resequencing of two replicate lines. Because the ITS sequence has 381 base pairs, we assumed the mutation rate to be *u* = 2.08 × 10^−10^ × 381 = 7.9 × 10^−8^ per generation. The generation time of an alga corresponds to the doubling time of algal population size. On the Greenland Ice Sheet, doubling times have been estimated as 3.75–5.5 days [52], while Onuma et al. [53] estimated the growth rate per day to be 0.42, which leads to a doubling time of (log 2)/0.42 = 1.65 days. Algae grow only during the period when snow melts and the duration depends on both altitude and latitude, but a typical duration of algal growth is ~2 months. Taking the reciprocal as the effective doubling time yields 11–36 days. Therefore, we assumed the generation time (annual mean) to be 24 days.

## Results

### Classification of snow algae in the ice core based on ITS2 sequences

We used high-throughput sequencing to obtain DNA sequences of algae from 19 layers of an ice core drilled on a glacier in central Asia, dated from present time to 8000 years ago (Fig. 1 and Table S1). In total, 17,016 unique sequences (phylotypes) for the fast-evolving algal nuclear rDNA internal transcribed spacer 2 (ITS2) region were determined in the ice core, from which 290 OTUs were defined with ≥98% nt sequence identity among all OTUs.

The ITS2 sequences were classified at the species level according to the genetic species concept based on secondary structural differences in the ITS2 region, which correlate with the boundaries of most biological species [38]. The ITS2 sequences from ice core samples were classified into 24 subgroups consisting of 17 chlorophycean, 5 trebouxiophycean, and 2 ulvophycean groups based on their secondary structures and BLASTn results (Fig. S1 and Tables S3–S4). The 17 subgroups of Chlorophyceae were subdivided into 10 subgroups of the *Chloromonadinia* clade, 1 subgroup of the *Monadinia* clade (recently treated as the genus *Microglena* [54]), 3 subgroups of the *Reinhardtinia* clade, 2 subgroups of the *Stephanosphaerinia* clade, and 1 subgroup corresponding to an unnamed group (which is related to *Ploeotila* sp. CCCryo 086-99) (for the clade names, see [55]). Although the *Chloromonadinia* clade contains several snow species belonging to *Chloromonas* or *Chlainomonas*, the 10 subgroups of the *Chloromonadinia* clade were considered to be *Chloromonas*. The 5 trebouxiophycean subgroups were composed of 2 subgroups of the *Chlorella* group, 1 subgroup of the *Raphidonema* group, 1 subgroup of the *Trebouxia* group, and 1 subgroup of the *Neocystis* group. The 2 subgroups of Ulvophyceae were closely related to the genus *Chamaetrichon* and *Planophila*, respectively. It is noted that *Sanguina* (‘*Chlamydomonas*’-snow group B [6]), *Ancylonema*, and *Mesotaenium*, which are snow algal genera found throughout the world [56, 57], were not detected in the ice core samples (Tables S3–S4).

### Global distribution of the *Raphidonema* group

**Fig. 2:**
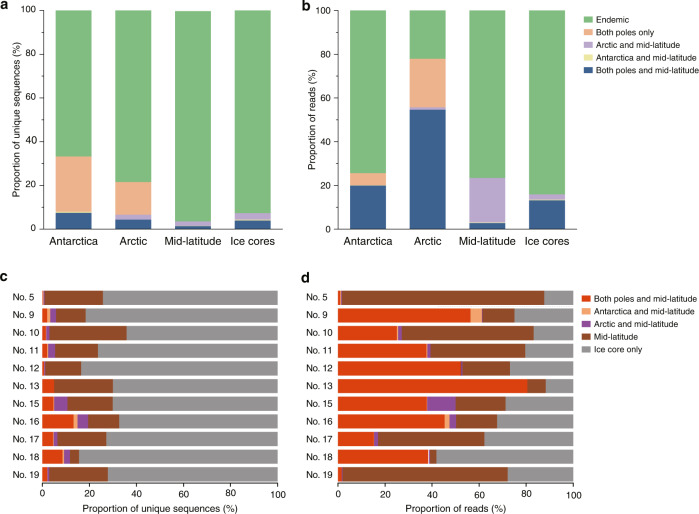
Distribution types of the Raphidonema group obtained from each region and the ice core based on ITS2 unique sequences. Proportions of unique sequence and number of sequencing reads are shown. **a** Unique sequences from surface snow and ice-core samples. **b** Number of sequencing reads from surface snow and ice core samples. **c** Unique sequences from the indicated locations within the ice core. **d** Number of sequencing reads of the unique sequences from the indicated locations within the ice core.

To understand the process by which snow algae form geographically specific population structures and how they migrate globally across the glaciers and snow fields, it is necessary to focus on the microbial species that inhabit the global cryosphere. Previous work elucidated that the *Raphidonema* group and ‘*Chlamydomonas*’-snow group B (*Sanguina*) are the cosmopolitans at both poles [6], but the latter was not detected in ice core samples examined in this study. Therefore, to elucidate the evolutionary history of the *Raphidonema* group, we further analyzed the ITS2 sequences obtained from the ice core sample as well as the glacier-surface samples from both poles [6] and from the mid-latitudes (samples from 10 sites, obtained in this study) (hereafter, surface samples; Table S2). Members of the *Raphidonema* group were detected in the older (deep core) layers of the ice core and at the glacier surface of central Asia (Fig. S1 and Tables S3–S4), as well as in the red snow samples from both poles [6]. In central Asia, the *Raphidonema* group was found in the Russian, Chinese, and Kyrgyz samples but was not detected in the Japanese and Tajik samples (Fig. S1 and Tables S3–S4). Combining these sequences yielded 893,649 reads and 22,389 unique sequences for subsequent detailed analysis (Tables S5–S6). The taxonomic composition of the *Raphidonema* communities differed among the mid-latitude, ice core, Arctic, and Antarctic samples as determined by PERMANOVA (Table S7). Most of the unique sequences in the *Raphidonema* group were consistent with an endemic distribution (Tables S8–S10). An average of 77% of the unique sequences of the *Raphidonema* group were endemic to a specific region (mid-latitude, 96%; Antarctic, 66%; Arctic, 79%), accounting for 40% of the total sequencing reads (mid-latitude, 77%; Antarctic, 74%; Arctic, 22%) (Fig. 2a, b and Tables S9–S10). This result suggested that most of the unique sequences are endemic, indicating that their dispersal has been limited to their respective regions [58–61].

Next, we analyzed the global distribution of the cosmopolitan phylotypes of the *Raphidonema* group, because a previous study analyzed their distribution only at the poles [6]. Only a limited number of unique sequences were distributed in all regions (mid-latitude, 1.4%; Antarctic, 5.6%; Arctic, 3.1%), accounting for a large proportion of the sequencing reads in polar regions but for only a small proportion in the mid-latitudes (mid-latitude, 2.8%; Antarctic, 20%; Arctic, 55%) (Figs. 2a, b, S2–S3, and Tables S9–S10). The distribution types of the *Raphidonema* group obtained from each region and the ice core were similar between the USEARCH and DADA2 analyses (Figs. 2, S4). In addition, we note that in ancient samples, post-mortem nt substitutions, such as cytosine to thymine, accumulate over many years of deposition [62], and these are not included in the DADA2 error model, which leads to the elimination of minor sequences in the DADA2 analysis. Therefore, we based our analysis on the results of the USEARCH unique sequences. These results suggested that only a few snow algae in the *Raphidonema* group were detected in samples from the mid-latitude regions.

Snow algae of the *Raphidonema* group were detected in different ice core layers, corresponding to different time periods. The ice core records revealed that the distribution types of the *Raphidonema* group have not changed significantly for the last 8000 years, with *p* = 0.1924 based on a PERMANOVA between the newer (1800–2001 AD) and the older (6000–8000 years before present) layers (Fig. 2c, d). In ice core samples, 77% of the unique sequences of the *Raphidonema* group were detected only in the ice core samples, accounting for 23% of the total sequencing reads (Fig. S5). Although some of these unique sequences may be artifacts of the post-mortem nt substitution or sequencing errors, because we conducted sequence quality filtering and removed the majority of artifact sequences by removing the singleton clusters, most of the unique sequences in the ice core are not likely to be artifacts, but they could represent endemic phylotypes (Figs. 2a, b, S5).

The cosmopolitan phylotypes were detected over a broad period as represented by ice core samples. They were present in approximately similar ratios in the newer and older layers (Fig. 2c, d). The cosmopolitan phylotypes were relatively abundant in the ice core samples (average, 4.0%; range, 0.2–13%), accounting for 13% (0.9–81% in the samples) of the total sequencing reads (Figs. 2c, d and S5).

### Microevolution of cosmopolitan and endemic phylotypes

We analyzed the evolutionary relationship between cosmopolitan and endemic phylotypes of the *Raphidonema* group among all snow surface and ice core samples. In total, 22,389 unique sequences of the *Raphidonema* group were clustered into 170 OTUs that were defined with ≥98% nt sequence identity among sequences within OTUs. The OTU sequences were subdivided into five subgroups (Groups A–E) based on phylogenetic analysis (Figs. S6–S11 and Tables S11–S12). Based on a previous study [63], Groups A–C and Group E were assigned to *R. sempervirens* and *R. nivale*, respectively, but Group D was not consistent with any species examined in that study (Fig. S6).

The phylotypes were categorized into three subsets: the cosmopolitan phylotypes found at both poles and the mid-latitude regions; the multi-region phylotypes found in any two of the Antarctic, Arctic, and mid-latitude regions; and the endemic phylotypes found in only one of the three regions. Cosmopolitan phylotypes were found in Groups A, B, and C and accounted for 64.6% of the unique sequences. We then analyzed the dispersal of the three groups in detail.

**Fig. 3:**
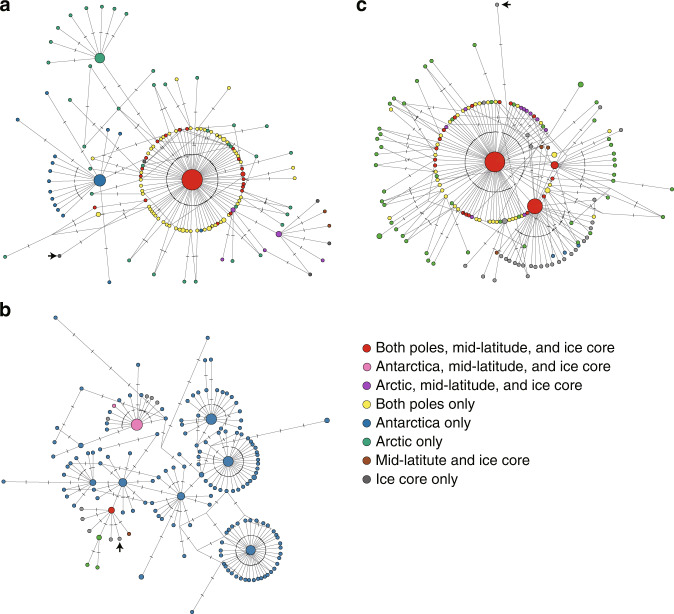
Phylogenetic relationships among phylotypes of the *Raphidonema* groups. Phylotype networks for ITS2 sequences within Groups A (**a**), B (**b**), and C (**c**) of the Raphidonema group that include the cosmopolitan phylotypes in this study. The median-joining method was used. Circles indicate phylotypes; the size of each circle is proportional to the number of unique sequences. Each notch on the edges represents a mutation. Phylotypes are colored according to geographic region. The arrow represents the phylotype in the outgroup (see Fig. S6).

MJ networks [47] for the ITS2 sequences in each subgroup revealed that the cosmopolitan phylotypes were located at the center of the networks in Groups A and C that contained any types (endemics, multi-regions, and cosmopolitans) of the phylotypes, whereas the endemic phylotypes were considered to be derived from the cosmopolitan phylotypes (Figs. 3 and S12–S13). Moreover, the outgroup phylotypes were directly connected to the cosmopolitan phylotypes. These findings clearly showed that the cosmopolitan phylotypes were ancestral, whereas the endemic phylotypes were derived. In contrast, there were remarkable differences in the shape of the networks between Group B and the others (Groups A and C). In Group B, the Antarctic endemic phylotypes formed a distinct clade, and multi-region phylotypes seemed to be recently derived from this clade. In addition, the Arctic endemic phylotypes formed another distinct clade. These two Group-B clades split directly from a cosmopolitan phylotype (5.3% of the total sequencing reads). For Groups A and C, however, major portions of the total sequencing reads belonged to cosmopolitan phylotypes in Groups A (48.2%) and C (62.4%), and the endemic and multi-region phylotypes were directly connected to these major cosmopolitan phylotypes in a radial manner—the so-called “star-like” pattern [64]. These contrasting network shapes seem to have been formed as a consequence of the unique evolution of each of these groups. We also found that sequences from ice cores did not represent a basal position (Figs. 3 and S12–S13). This is because the haplotypes found in the modern samples have existed from times earlier than the ice core ages, due to the very small mutation numbers expected to have occurred since the ice core ages. Therefore, detected ice core ages were not included in the molecular evolution calculations of our demographic model. However, the phylogenetic networks themselves do not provide information on the evolutionary time scale. Hence, the ice core samples provide further direct evidence that *Raphidonema*, especially cosmopolitans belonging to this genus, persistently grew on snow and ice at least during the Holocene, and their ITS2 sequences have not changed over the last 8000 years.

**Fig. 4:**
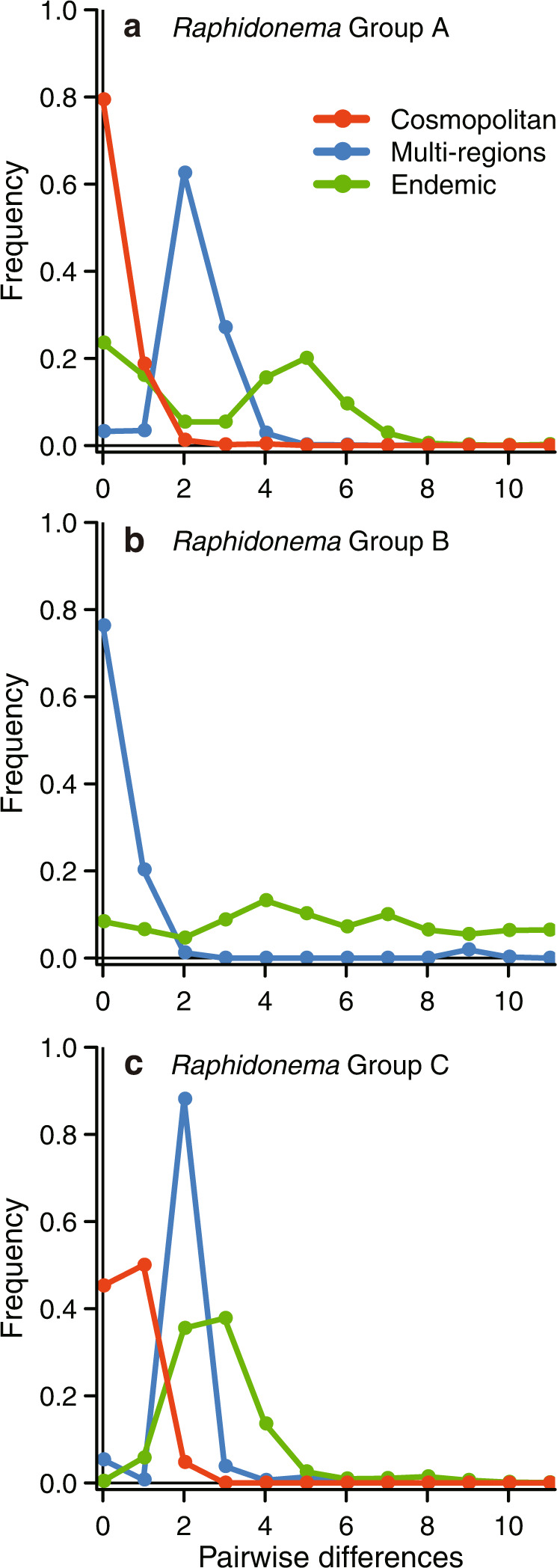
Mismatch distribution based on the number of pairwise differences in each distribution type in *Raphidonema* groups. The lines represent the observed number of pairwise differences in each distribution type (cosmopolitan, multi-region, endemic) within the *Raphidonema* Groups A (**a**), B (**b**) and C (**c**). Calculations were performed for all distribution types of *Raphidonema* Groups A and C, for which various cosmopolitan phylotypes were detected. On the other hand, calculations for only multi-region and endemic phylotypes were performed for *Raphidonema* group B, because no variation was found in cosmopolitan phylotypes.

Referring to “ancestral” phylotypes as those having a longer history than other, more recently derived phylotypes, it is possible that individuals not closely related can share the same ancestral phylotype. In such cases, if genetically far-related individuals from various geographical regions share the same ancestral phylotype, they appear to be “cosmopolitan” (Fig. S14a). In order to distinguish between these “apparent cosmopolitans”, and “true cosmopolitans” that migrate globally, it is necessary to show that the cosmopolitan and endemic phylotypes have distinct demographic histories rather than being part of a continuous population sharing certain demographic dynamics (Fig. S14). Because phylotype networks are not useful for quantifying the rate(s) of microevolution, we used the coalescent model to quantify phylotype demographics [65]. As numerous phylotypes must be analyzed with this approach, we concentrated on statistical inference based on pairwise comparisons of phylotypes, for which the likelihood can be determined in a practical manner (see Materials and Methods). Histograms for the number of mismatched sites between two phylotypes chosen from a set of phylotypes, which will be called the pairwise mismatch distribution, are shown in Figs. 4 and S15. For Groups A and C, the distribution among cosmopolitans, multi-regions, and endemics was unimodal, in which the modes align from left to right with the order cosmopolitans, multi-regions, and endemics. Rogers and Harpending [48] noted that this “wave” propagation results from the expansion in size of a population, which leads to large mismatches, and the mode shifts to the right (see Fig. 2 of [48]). As time passes, the mode shifts to the left and eventually returns to the origin, i.e., representing a population that has not undergone an expansion event. Rogers and Harpending obtained an approximate solution for the wave and fitted the solution to human mitochondrial sequence data. We improved upon their method based on the coalescent model (see Materials and Methods) and applied it to the ITS2 sequence data for snow algae.

For Group A, when we fit the single demographic model to all phylotypes, the log-likelihood was –414,487. In contrast, when we fit the demographic model to each subset, that is, cosmopolitans, multi-regions, and endemics, separately, the log-likelihood was –341,964. Because the latter is larger than the former, we fit the model to each subset of phylotypes separately. For Group C, when we fit the demographic model to the cosmopolitans, multi-regions, and endemics separately, the log-likelihood was –142,106, which is larger than the log-likelihood, –218,080, when we fit the single demographic model to all phylotypes. In contrast to Groups A and C then, we fit the single demographic model to all phylotypes of Group B because the log-likelihood, –196,070, was larger than the log-likelihood, –220,145, when we fit the demographic model to the cosmopolitans, multi-regions, and endemics separately. These results suggested that cosmopolitans, multi-regions, and endemics experienced different demographic histories in Groups A and C, whereas they had the same demographic history in Group B (Table S13). These results indicate the cosmopolitans in Group A and C are true cosmopolitans, whereas the those in Group B can be regarded as an apparent cosmopolitan.

The ML estimates of $\tau = 2ut_0$, $\theta _0 = 2N_0u$, and $\theta _1 = 2N_1u$ are shown in Table S13 with standard deviation values. The population expanded *t* years ago, with the size before and after the expansion being represented by *N*_0_ and *N*_1_, respectively. The mutation rate (*u*) was assumed to be 7.9 × 10^–8^/ sequence/generation, and the generation interval was assumed to be 24 days (Materials and Methods). In Group A, for the cosmopolitans, the estimates of *t, N*_0_, and *N*_1_ were $33.8/(2 \times 7.9) \times 10^8 \times {\textstyle{{24} \over {365}}} = 1.4 \times 10^7$ years, $(0.108 - 0.010)/(2 \times 7.9) \times 10^8 = (6.8 - 0.63) \times 10^5$, and $(0.217)/(2 \times 7.9) \times 10^8 = 1.4 \times 10^6$, respectively. In the same way, we computed estimates of *t, N*_0_, and *N*_1_ of other phylotypes and other groups (Table S14). For the endemics, the respective values were 9.2 × 10^6^ years, 80, and 2.1 × 10^7^, and the values were 4.6 × 10^6^ years, 139, and 1.5 × 10^7^ for the multi-regions. Taking into account the minimum and maximum ranges of the mutation rates per generation as well as the generation intervals, *t* for cosmopolitans was 3.6 × 10^6^–4.0 × 10^7^ years ago, and *t* for endemics was 2.3 × 10^6^–2.6 × 10^7^ years ago (Table S14). These results suggested that the cosmopolitans existed at least 1.4 × 10^7^ years ago, and the endemics were derived from the cosmopolitans 9.2 × 10^6^ years ago. The size of the endemics expanded 2.6 × 10^5^-fold, which may have resulted from extensive dispersal. The multi-regions tended to mimic the endemics. Note that our demographic model was simplified to avoid overparameterization. In reality, considering the branching patterns of the MJ network, it is plausible that the endemic phylotypes have been repetitively and continuously derived from the cosmopolitans in multiple lineages—from 9.2 × 10^6^ years ago to the present. In the same way, as for Group C, our results suggested that the cosmopolitan population expanded 3.9-fold ~3.2 × 10^6^ years ago, and the endemics were derived from the cosmopolitans 1.9 × 10^5^ years ago. The size of the endemics expanded 59-fold. In contrast to the phylotypes of Groups A and C, those of Group B experienced no significant expansion (Supplementary Results). In Groups A and C, the derived endemics (and multi-regions) expanded greatly as compared with the ancestral cosmopolitans (Table S14). These extraordinary expansions constitute evidence for local adaptation by the endemic/multi-region populations. In contrast, there was no evidence of local adaptation in Group B. The mismatch distribution of the entire Group B (multi-regions + endemics) showed a multimodal pattern (Fig. 4), which is present in the populations with stable sizes for a long period. When the populations finally reach equilibrium, the mismatch distributions show the exponential distribution [48]. Based on our ML estimates (Table S14), the historical population of Group B has been stable.

## Discussion

Our study provides substantive insight into the global microbial dispersal process, which has been controversial for more than a century [1]. The ongoing regional expansion of snow algae is likely to be affected substantially by global climate change because the cryosphere is particularly sensitive to the climate. Our results demonstrate that the current microbial distribution was maintained throughout the Holocene, i.e., at least for the last 8000 years, based on the analysis of ancient DNA from ice core samples. Furthermore, our inference of the demography of snow algae suggests that local adaptation of the endemic phylotype of snow algae occurred millions of years ago rather than after the last glacial period. Microorganisms residing in snow and ice were affected by variations in the extent of the cryosphere associated with climatic changes such as glacial-interglacial cycles. However, the detailed association between environmental fluctuations and the evolution of microorganisms in the cryosphere remains unknown. Therefore, elucidating this association will shed more light on the evolution of microorganisms that populated snow and ice in past glacial cycles.

Our results reveal the existence of cosmopolitan phylotypes, which have dispersed globally and regionally to form endemic phylotypes. Although the *Raphidonema* group makes up the majority of present-day cosmopolitan species [6], details pertaining to their phenotypic variations and life cycles remain unknown. Two main explanations have been given for how snow algae disperse globally, namely by aerial transport and by animal transport (e.g., via fecal droppings) [4]. Based on field surveys and laboratory experiments [66, 67], vegetative cells of *Raphidonema* can be transferred by strong winds and dispersed via atmospheric circulation. Chlorophycean snow algae (e.g., *Sanguina* and *Chloromonas*) generally produce zygotes or cysts to become dormant with resistance to desiccation and possibly to UV radiation. Such zygotes/cysts are suitable for long-distance dispersal; however, zygote formation of *Raphidonema* was reported only once in 1953 [68], but has not been observed in subsequent studies using field-collected and cultured materials (e.g., [69, 70]). Therefore, further research is necessary to understand the mechanism by which microorganisms are dispersed via the atmosphere and how they ultimately accumulate in the global cryosphere, as well as any potential effects of UV radiation.

A previous study has suggested that snow species of *Raphidonema* (*R. sempervirens* and *R. nivale*) are actually soil algae that are only occasionally transported to snow [66]. This appears to be similar to some terrestrial microalgae, such as *Chlorella* and *Stichococcus* (Trebouxiophyceae), which have broad temperature ranges for growth and are also found on snow/glacier surfaces. We note, however, that the optimum growth temperatures for both species are below 6 °C, and *R. sempervirens* cannot grow above 13 °C [71]. In addition, the temperature ranges for growth for *R. nivale* and *R. tatrae* are 0–15 °C and 0–10 °C, and the optimum temperatures are 5 and 4 °C, respectively [72, 73]. Therefore, if these species were truly soil algae, their habitats would likely be restricted to cold environments, such as alpine and polar regions. Recent amplicon sequencing analysis for soils from the French Alps reported that *Raphidonema* spp. were rarely detected from soils at high elevations, although the samples contained enough *Sanguina* spp., a well-known genus of snow algae that are associated with red snow, to be detected [74]. Therefore, a large proportion of *Raphidonema* sequences in the surface samples indicated that the snow species of the genus probably grow on snow and ice rather than simply being transported from the periglacial soils.

Although *Raphidonema* is reported to be a minor genus as compared with other snow/glacier algae (e.g., *Ancylonema*, *Mesotaenium*, and *Sanguina*) on modern snow surfaces [75], the latter “dominant” snow/glacier algae were not detected in the ice cores analyzed in this study. According to the altitudinal distribution of phototrophs on a glacier in Alaska [76], *Raphidonema* is categorized as an opportunist, appearing on the surface under certain conditions. The ice core samples analyzed in this study were from central Asia, where the pH of glacial meltwater is higher than in other areas due to abundant carbonates derived from nearby deserts [77]. Under these conditions, cyanobacteria are most dominant, and only a few green algae grow on such glaciers (e.g., [78]). According to Hoham and Remias [75], snow green algae are usually found in snow with an acidic pH. We believe that this higher pH is probably one reason for the absence of green algae other than *Raphidonema* in this area, although future studies are needed to confirm this point.

In conclusion, we examined the genetic structures of snow algae from both poles and mid-latitude high-mountain regions to determine the history of biogeographical distributions and evolutionary relationships between cosmopolitan and endemic phylotypes of snow algae. We found that the genus *Raphidonema* (trebouxiophycean group) was distributed over both poles and mid-latitude regions and was detected in different ice core layers, corresponding to distinct time periods. A phylogenetic network analysis consistently showed that the cosmopolitan phylotypes were located near the root positions, whereas the endemic phylotypes were generally located at the tips, suggesting that the endemic phylotypes originated from the cosmopolitan phylotypes. To examine this hypothesis, we applied a coalescent theory–based demographic model to our data, which confirmed the better fit of an evolutionary scenario with the assumption that the cosmopolitan and endemic phylotypes experienced different population histories, as compared with a scenario with the assumption that these phylotypes shared common population histories. The evolutionary parameters estimated using this model indicated that the modern cosmopolitan phylotypes were present long before the last glacial period (Supplementary Results, Table S14) and that the endemic phylotypes originated from the ancestral cosmopolitan phylotypes. These findings suggest that the cosmopolitans dispersed worldwide, from which were derived new localized endemics.

Although the local adaptations of endemics were inferred from their demographic histories, a precise picture of how newly derived endemics have dispersed and adapted to local environments and of the process by which they have migrated across the global cryosphere is still unclear. Future work will benefit from single-cell genomic technologies to shed light on their morphological and metabolic aspects and will provide new insights into their dispersal and adaptive evolution.

## Supplementary information

Supplemental Information

## Data Availability

The raw sequence data are available under DDBJ DRA ID DRA012482. The nucleotide alignments of 98% sequence clustering of unique ITS2 sequences are available from http://redsnow2021.paleogenome.jp/.
