# Supplementary material for: Evolution of snow algae, from cosmopolitans to endemics, revealed by DNA analysis of ancient ice
Source: ISME J. 2023 Jan 17;17(4):491–501. doi: 10.1038/s41396-023-01359-3 (PMC10030584; doi:10.1038/s41396-023-01359-3)
Supplement: Supplementary file 1 — Supplemental Information [file 41396_2023_1359_MOESM1_ESM.pdf]

Supplemental Information

Evolution of snow algae, from cosmopolitans to endemics, revealed by DNA analysis of ancient ice

Takahiro Segawa, Takahiro Yonezawa, Ryo Matsuzaki, Hiroshi Mori, Ayumi Akiyoshi, Francisco Navarro, Koji Fujita, Vladimir B. Aizen, Zhongqin Li, Shuhei Mano, Nozomu Takeuchi

This PDF file includes:

Supplementary Results

Supplementary Figures S1–S16

Supplementary Tables S1–S14

Supplementary Results

Demographic analysis

Demographic parameters (t, N_0, and N_1) are summarized in Table S14. The tMRCAs (time of the most recent common ancestors) were also calculated as $2 \times N_1$ generations ago under the assumption of the haploidy populations (if t years > $2 \times N_1$ generations). If $2 \times N_1$ generations > t years, as the coalescent process under the constant population size no longer holds, we assumed tMRCA as $t + 2 \times N_0$ generations ago.

Taking into account the 95% confidence interval of the mutation rate (1.09–3.74 × 10^{-10}/site/year; [S1]) as well as the range of the generation interval (11–36 days; [S2, S3], the minimum–maximum range of the mutation rate was assumed to be 4.21×10^{-7} to 4.73×10^{-6}/sequence/year. The ranges of the demographic parameters (t, N_0, N_1, and tMRCA) were also estimated within this framework.

As mentioned in the main text, endemics were derived from the cosmopolitans $1.9 \times 10^5 – 9.2 \times 10^6$ years ago in Groups A and C, corresponding to the Late Miocene to the Middle Pleistocene. A global cooling event began in the Late Miocene and has continued to the present day, and it characterizes this period through the Cenozoic Era in terms of the inception of ice-sheets in both hemispheres [S4]. It is possible that environmental diversity in the cryosphere increased during this period, and this may have increased the prevalence of endemism as a result of local adaptations.

We also estimated the demographic histories of the cosmopolitans and endemics for each of Groups A, B, and C based on the Bayesian Skyline Plot [S5] with BEAST ver. 1.10.4 [S6]. The drift of the molecular evolutionary rate was modeled by the uncorrelated log-normal relaxed (UCLD) clock. The UCLD mean was assumed to be $2.08 \times 10^{-10} \times (365/24) = 3.16 \times 10^{-9}$/site/year. The standard deviation of the UCLD mean was calculated from the combinations of the minimum–maximum range of the mutation rate per generation [S1] and the generation

interval [S2, S3] and was assumed to be 4.94×10^{-9}/site/year. A normal distribution was assumed and truncated to 1.24×10^{-8}/site/year for the upper boundary and 1.11×10^{-9}/site/year for the lower boundary. The HKY + I + Γ model was used for the nucleotide substitution model. MCMC was conducted under 200 million generations, and trees were sampled every 10,000 generations.

The tMRCAs estimated by the Bayesian Skyline Plot using BEAST are summarized in Table S14. tMRCAs estimated by BEAST and by the ML method were fundamentally consistent. Although the Bayesian Skyline Plot can flexibly estimate the demographic histories (e.g., expanding, contracting, stable), they can be traced back only to the tMRCA. In contrast, our ML method, which assumes an expanding or stable population size model, can capture the demographic signal before the tMRCA. Accordingly, the demographic histories estimated by the ML and BEAST were not directly comparable for Group A cosmopolitans and endemics and Group C cosmopolitans because of $t >$ tMRCA. However, as $t <$ tMRCA in Group C endemics, those results were comparable (Table S14). The demographic histories of the cosmopolitans and endemics for each of Group A, B, and C as estimated by the Bayesian Skyline Plots are shown in Fig. S16. The timing of the population size expansion in Group C was ~200,000 years ago for the ML method (51,000–570,000 years ago, considering the range of mutation rates and generation intervals). In contrast, BEAST also showed a significant population size increase ~300,000 years ago, and thus the two results are consistent.

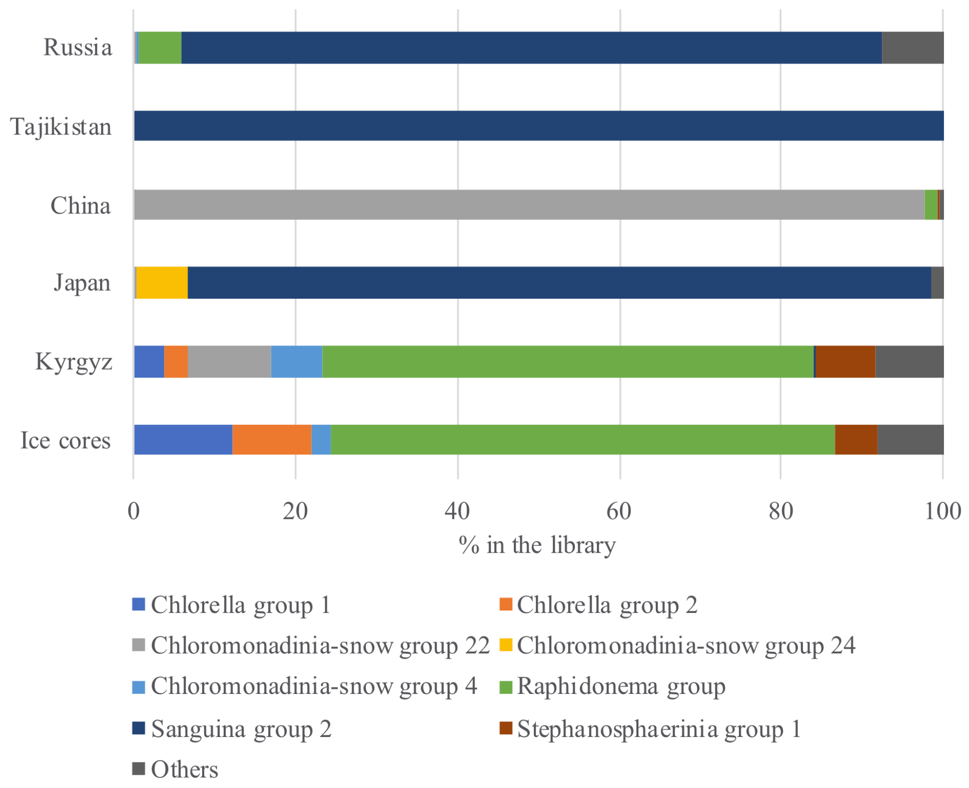

Figure S1: Algal taxonomic composition of the reads in snow and ice samples based on ITS2 sequences. The bar chart presents the average community composition for each region based on the five major ITS2 groups and low-abundance groups (grouped as Others).

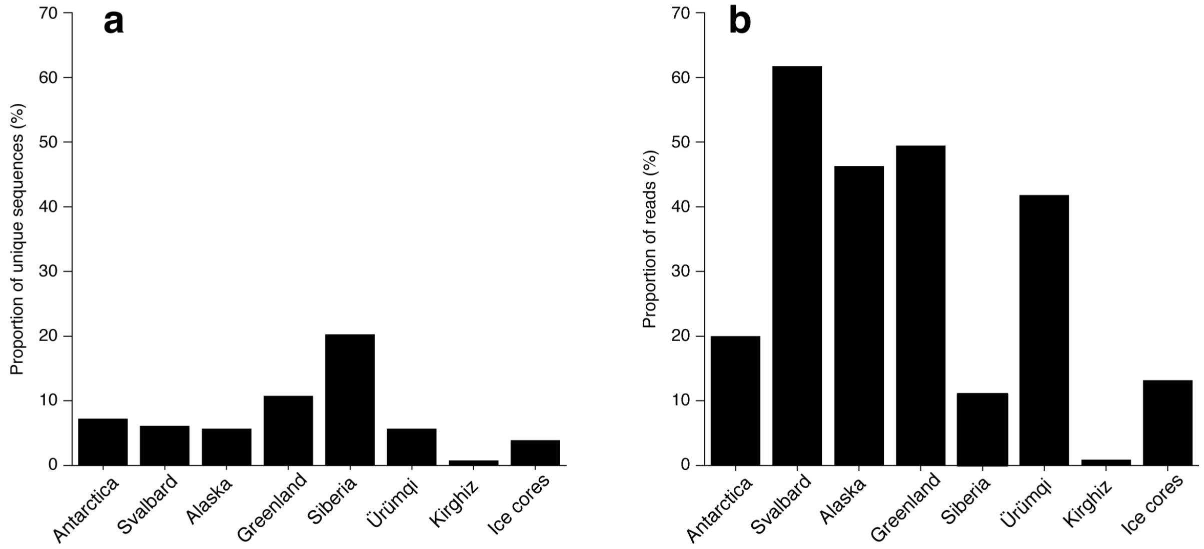

Figure S2: Distribution of the both poles and mid-latitude cosmopolitan in the *Raphidonema* group obtained from each region based on unique ITS2 sequences from high-throughput sequencing. Unique sequences and sequencing read numbers are shown. **(a)** Unique sequences. **(b)** Number of sequencing reads of the unique sequences.

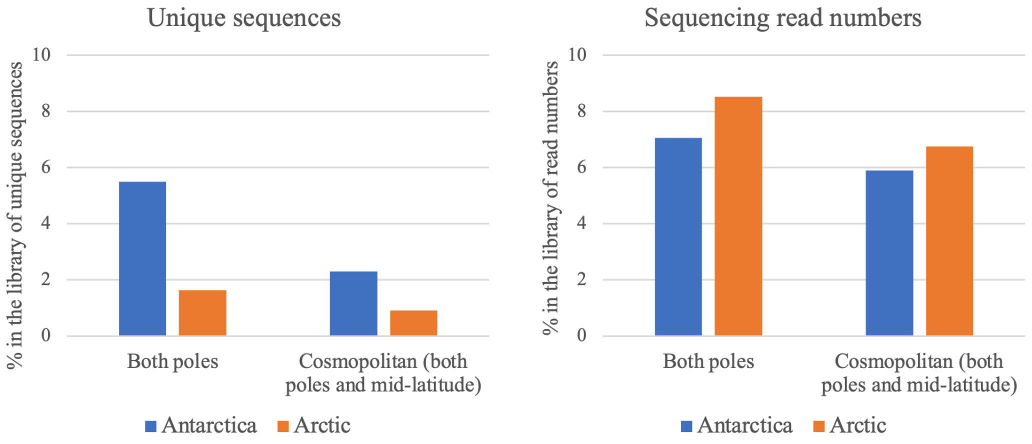

Figure S3: Distribution of the both poles (detected from Antarctica and the Arctic, namely Svalbard, Greenland, and Alaska) and cosmopolitan (both poles and mid-latitude) snow algae in the *Raphidonema* group obtained from each region based on unique ITS2 sequences. Unique sequences and sequencing read numbers are shown. **Left** Unique sequences. **Right** Number of sequencing reads. 56% unique sequences and 79.9% sequencing reads of the both poles distribution were observed in the cosmopolitan distribution (both poles and mid-latitudes).

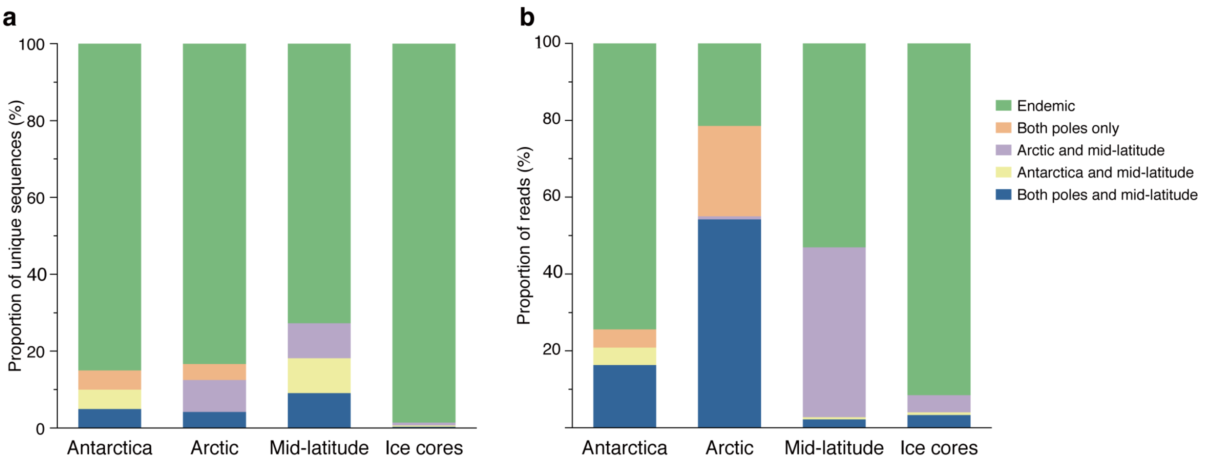

Figure S4: Distribution types of the *Raphidonema* group obtained from each region and the ice core based on ITS2 unique sequences by DADA2 analysis. **(a)** Unique sequences from surface snow and ice-core samples. **(b)** Number of sequencing reads from surface snow and ice core samples.

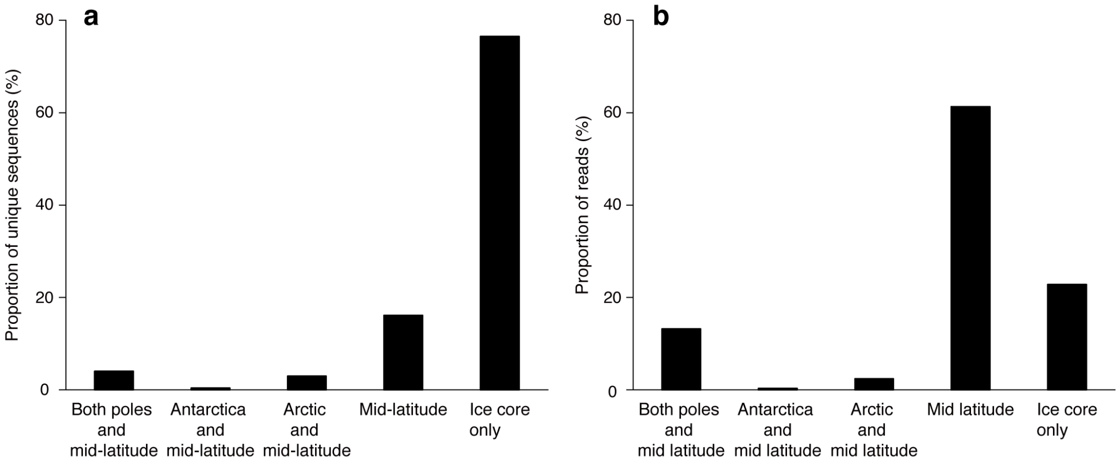

Figure S5: Average distribution categories in the *Raphidonema* group obtained from ice-core samples based on unique ITS2 sequences from high-throughput sequencing. Unique sequences and sequencing read numbers are shown. **(a)** Unique sequences. **(b)** Number of sequencing reads.

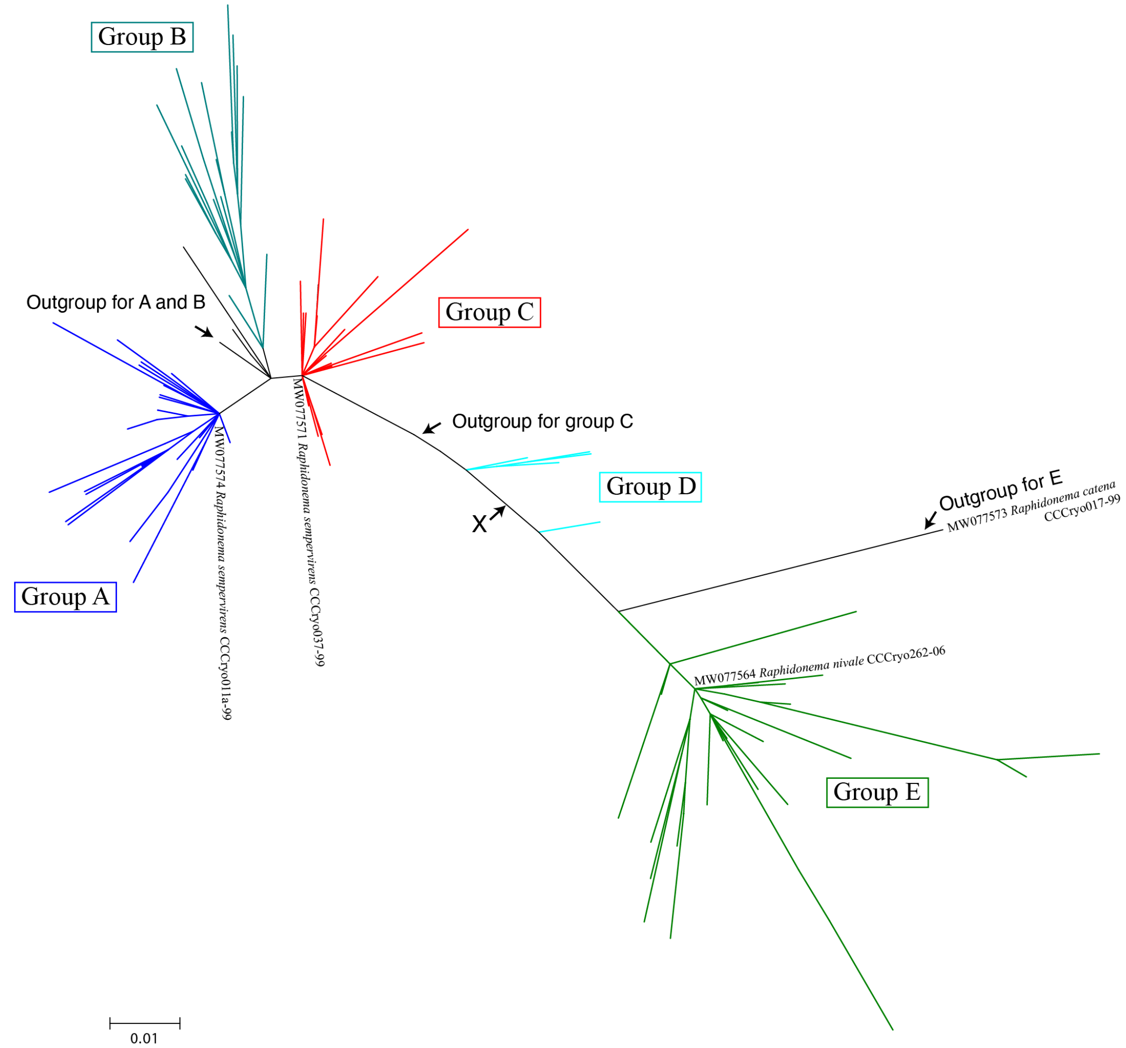

Figure S6: Phylogenetic relationship of 170 operational taxonomic units (OTUs) (98% OTU) in the *Raphidonema* group based on ITS2 sequences. Alignment among the OTUs was carried out based on ITS2 secondary structures. A maximum likelihood tree was constructed with 1000 bootstrap replications using IQ-TREE version 1.6.12 with the TVMe+G4 model. The groups are labeled and distinguished based on color. Detailed information about the sequences used for species identification is provided [S7]. The root position of this tree (indicated by '×') was assigned using the MinVar method [S8]. The MAD method [S9] showed essentially the same result. By locating this root position, the ancestral (closer to the root) and the derived (farther from the root) nodes can then be defined. The sequences derived from the closest ancestral node from each MRCA (most recent common ancestor) of Groups A–E was used for the outgroups for Groups A–E (Fig. 3, Fig. S12, S13).

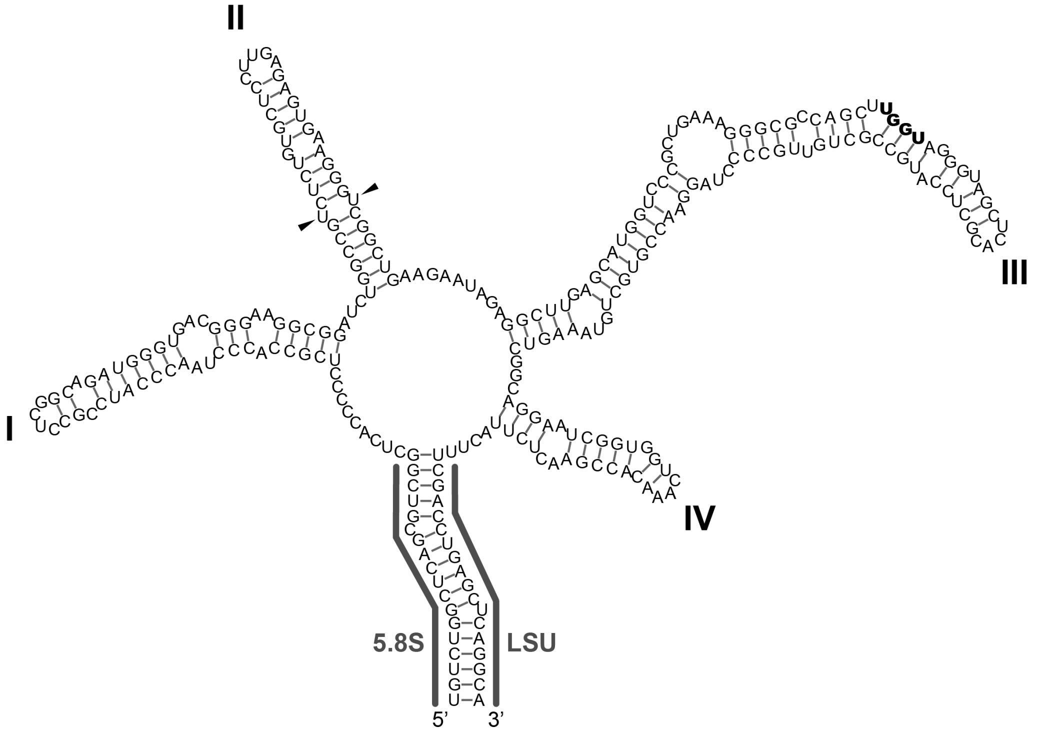

Figure S7: Predicted secondary structure of the nuclear rDNA ITS2 transcript of *Raphidonema* Group A (Unique sequence no. Uniq1360). The 3’ end of the 5.8S ribosomal RNA (rRNA) and the 5’ end of the large subunit of rRNA (LSU rRNA) are indicated by dark lines. Note the U-U mismatch in helix II (arrowheads) and the YGGY motif (UGGU) on the 5’ side of helix III (bold type).

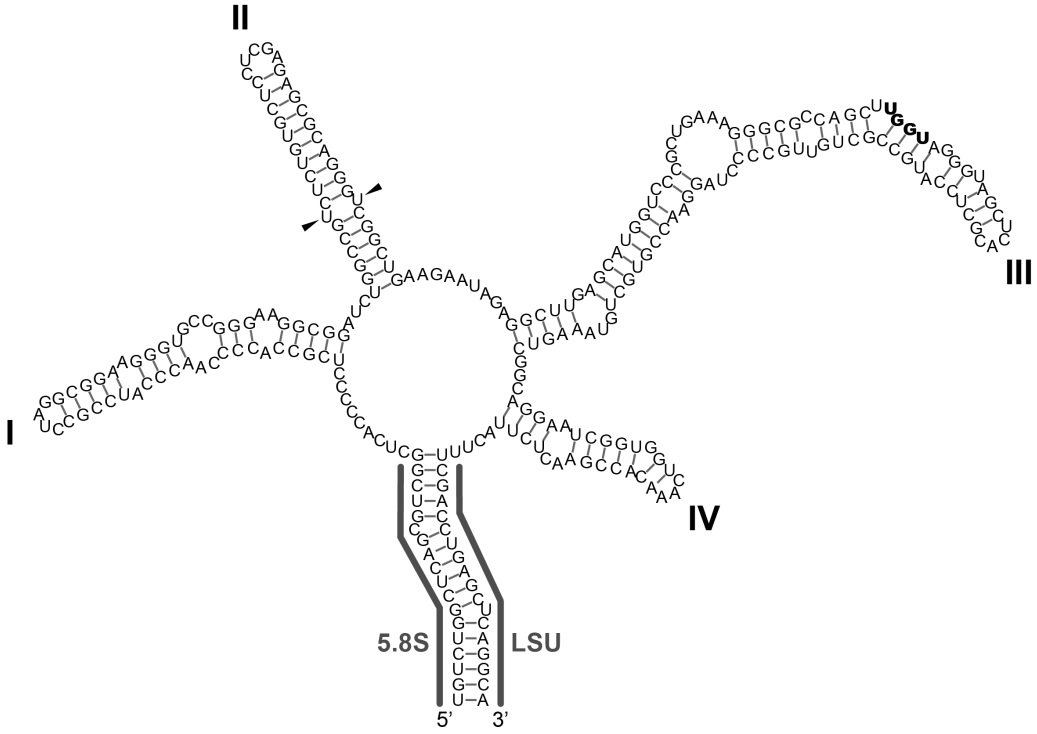

Figure S8: Predicted secondary structure of the nuclear rDNA ITS2 transcript of *Raphidonema* Group B (Unique sequence no. Uniq2588). The 3’ end of the 5.8S ribosomal RNA (rRNA) and the 5’ end of the large subunit of rRNA (LSU rRNA) are indicated by dark lines. Note the U-U mismatch in helix II (arrowheads) and the YGGY motif (UGGU) on the 5’ side of helix III (bold type).

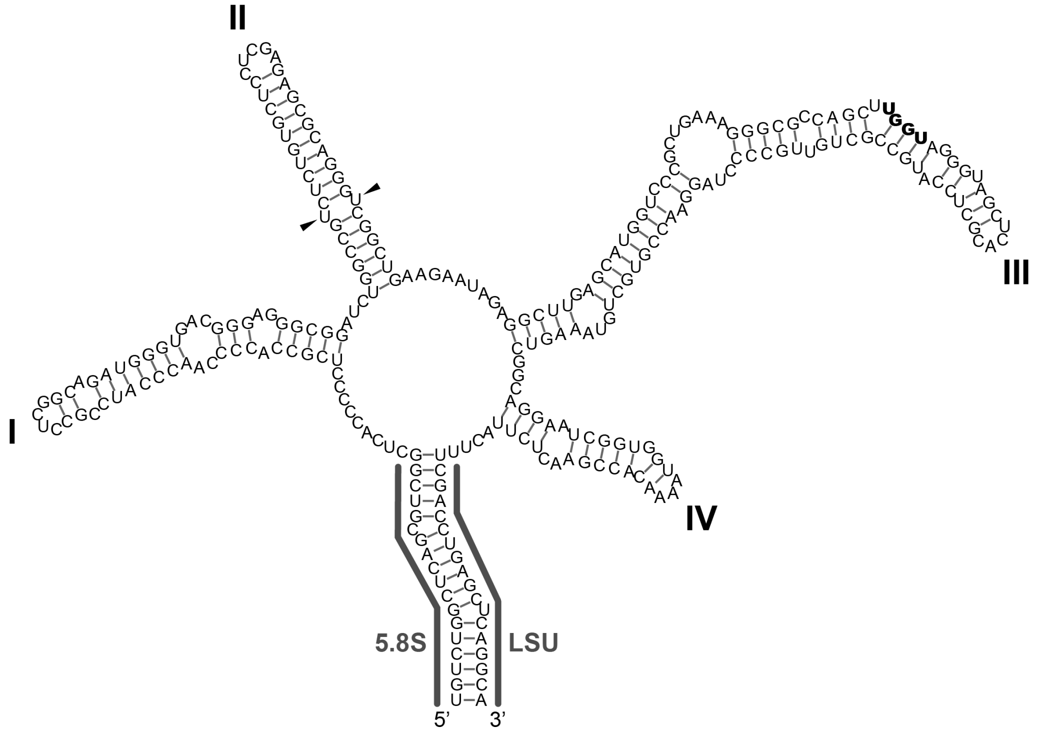

Figure S9: Predicted secondary structure of the nuclear rDNA ITS2 transcript of *Raphidonema* Group C (Unique sequence no. Uniq1782). The 3’ end of the 5.8S ribosomal RNA (rRNA) and the 5’ end of the large subunit of rRNA (LSU rRNA) are indicated by dark lines. Note the U-U mismatch in helix II (arrowheads) and the YGGY motif (UGGU) on the 5’ side of helix III (bold type).

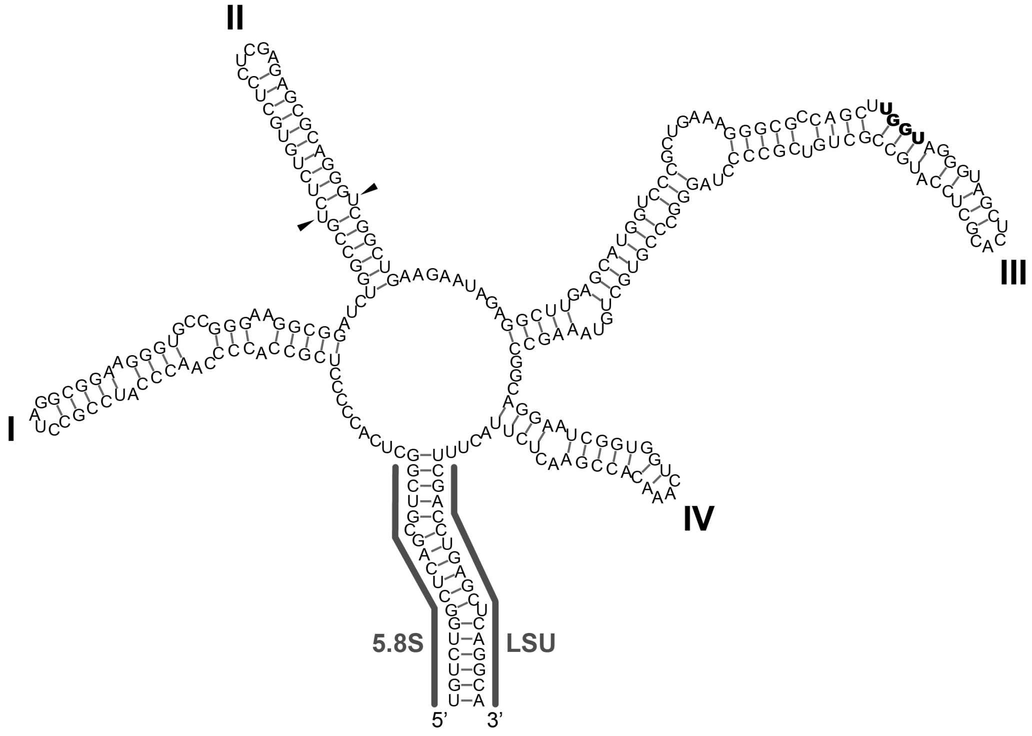

Figure S10: Predicted secondary structure of the nuclear rDNA ITS2 transcript of *Raphidonema* Group D (Unique sequence no. Uniq1367). The 3’ end of the 5.8S ribosomal RNA (rRNA) and the 5’ end of the large subunit of rRNA (LSU rRNA) are indicated by dark lines. Note the U-U mismatch in helix II (arrowheads) and the YGGY motif (UGGU) on the 5’ side of helix III (bold type).

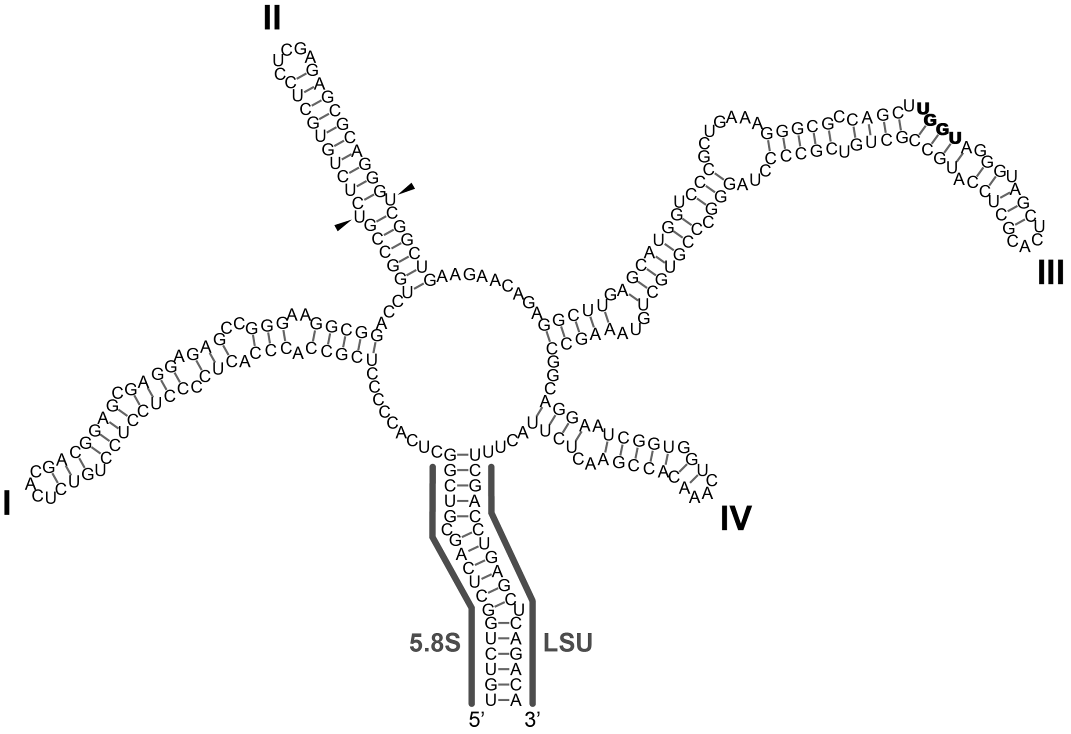

Figure S11: Predicted secondary structure of the nuclear rDNA ITS2 transcript of *Raphidonema* Group E (Unique sequence no. Uniq2918). The 3' end of the 5.8S ribosomal RNA (rRNA) and the 5' end of the large subunit of rRNA (LSU rRNA) are indicated by dark lines. Note the U-U mismatch in helix II (arrowheads) and the YGGY motif (UGGU) on the 5' side of helix III (bold type).

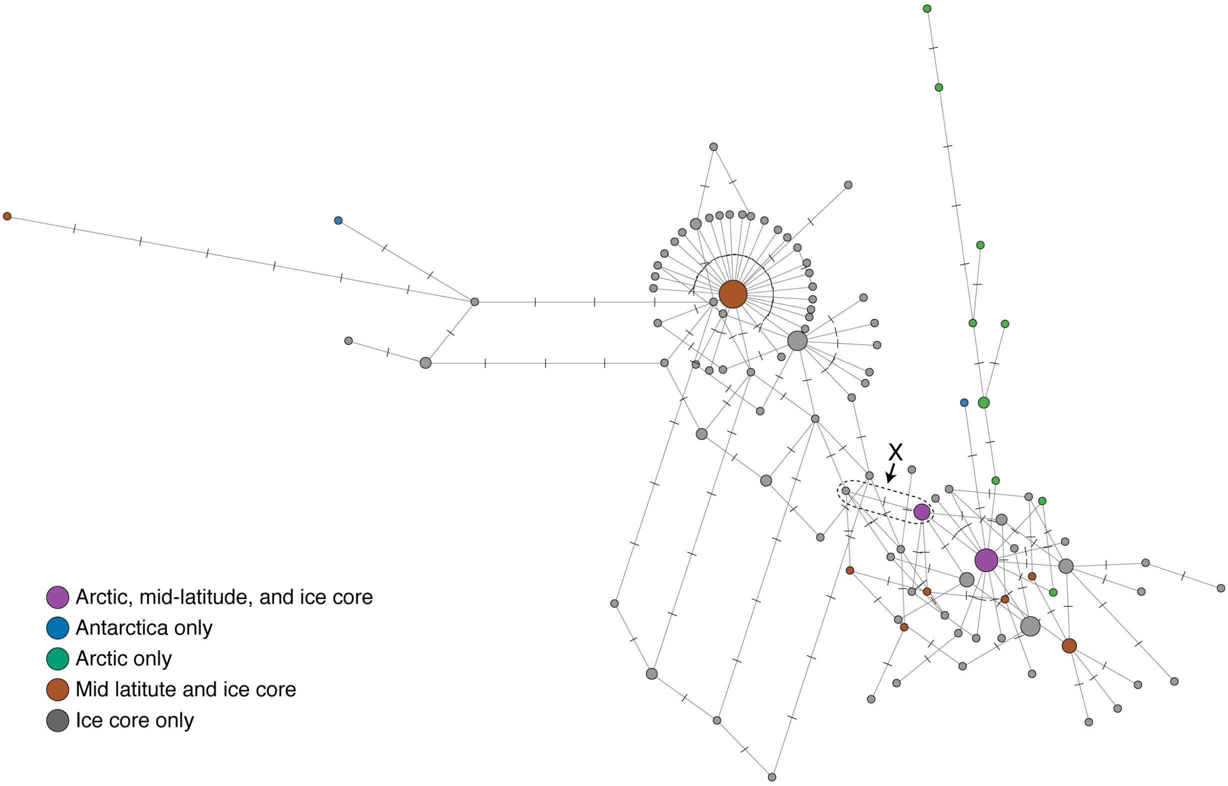

Figure S12: Phylotype networks for ITS2 sequences of *Raphidonema* Group D in this study. The median-joining method was used. Circles indicate phylotypes; the size of each circle is proportional to the number of unique sequences. Each notch on the edges represents a mutation. Phylotypes are colored according to geographic region. The area enclosed in a dashed-lined oval shape indicates the approximate position of the root in Figure S6.

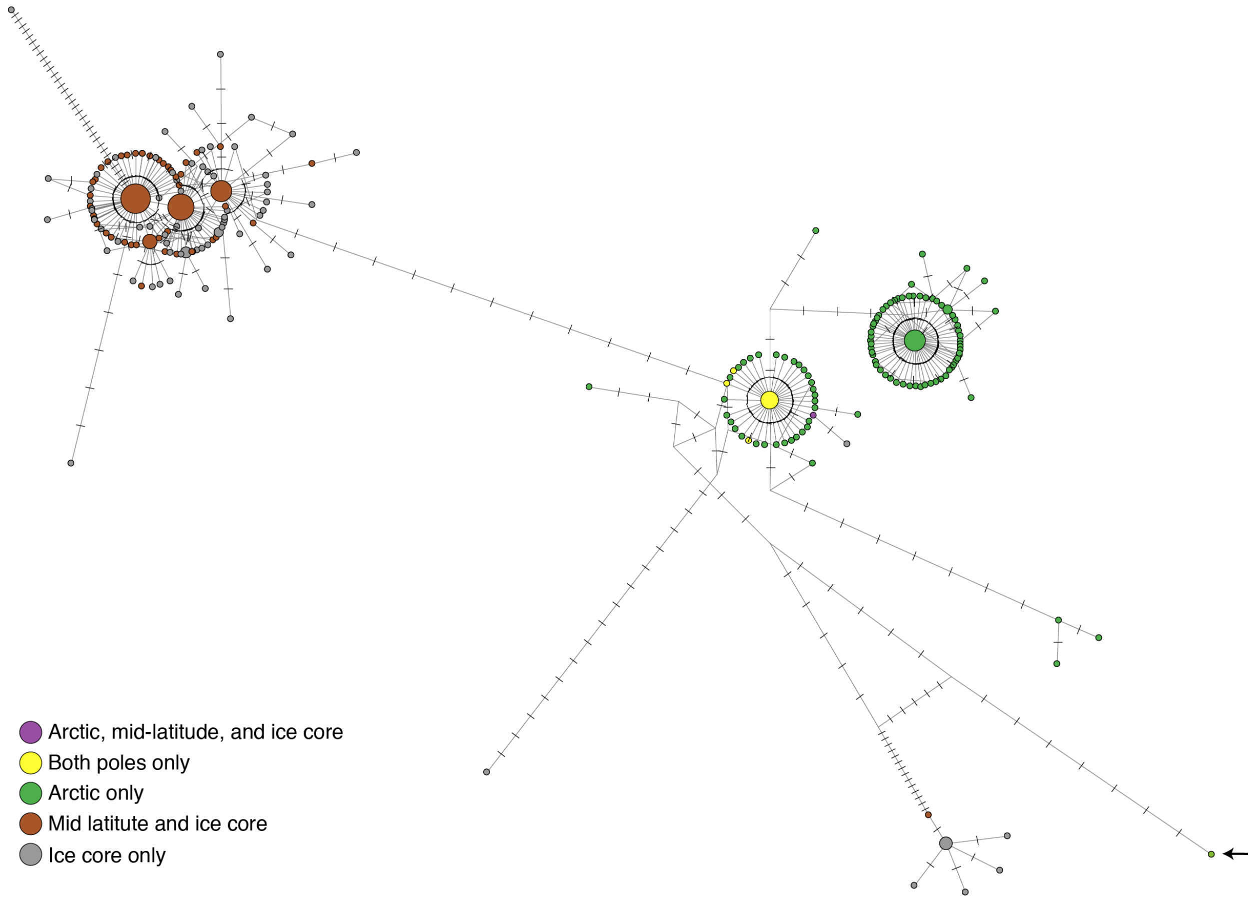

Figure S13: Phylotype networks for ITS2 sequences of *Raphidonema* Group E in this study. The median-joining method was used. Circles indicate phylotypes; the size of each circle is proportional to the number of unique sequences. Each notch on the edges represents a mutation. Phylotypes are colored according to geographic region. The arrow represents the phylotype in the outgroup.

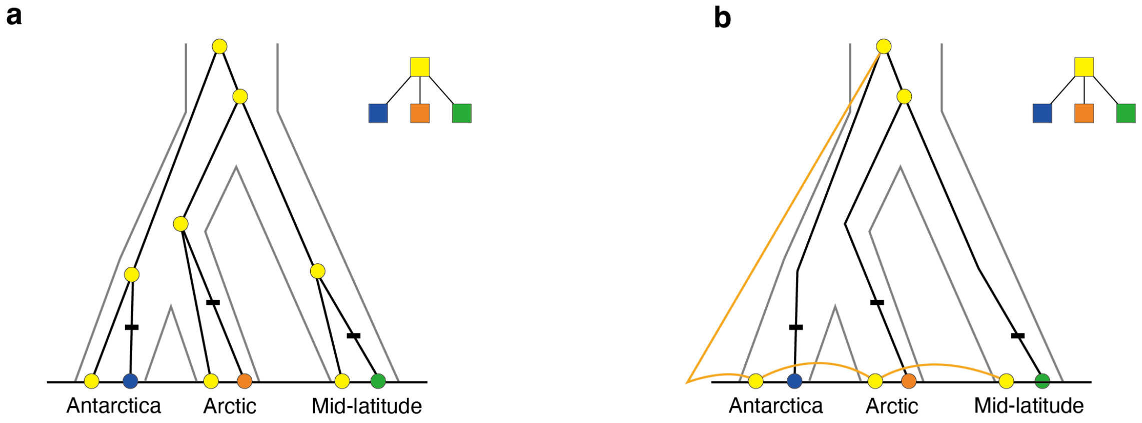

Figure S14: Conceptual diagram of "apparent cosmopolitan" and "true cosmopolitan". When the cosmopolitan phylotype is the ancestral type, any of the following two possibilities can occur: "apparent cosmopolitan" and "true cosmopolitan". **(a)** Apparent cosmopolitan. Genetically distantly related individuals share the same "ancestral" phylotype, detected in several different localities. **(b)** True cosmopolitan. Genetically closely related individuals move between regions within a short period (the geographic origin of true cosmopolitans is unknown). Black bold lines on the branches of the phylogenetic tree indicate mutations. Color points represent distribution type: cosmopolitans–yellow circles, endemics–blue, orange and green circles. If the ITS2 sequences lack sufficient phylogenetic information, the phylogenetic network of **(a)** and **(b)** can be the same in terms of haplotype divergence patterns (**a**, **b** upper right). Therefore, these two possibilities cannot be distinguished merely from the phylogenetic network.

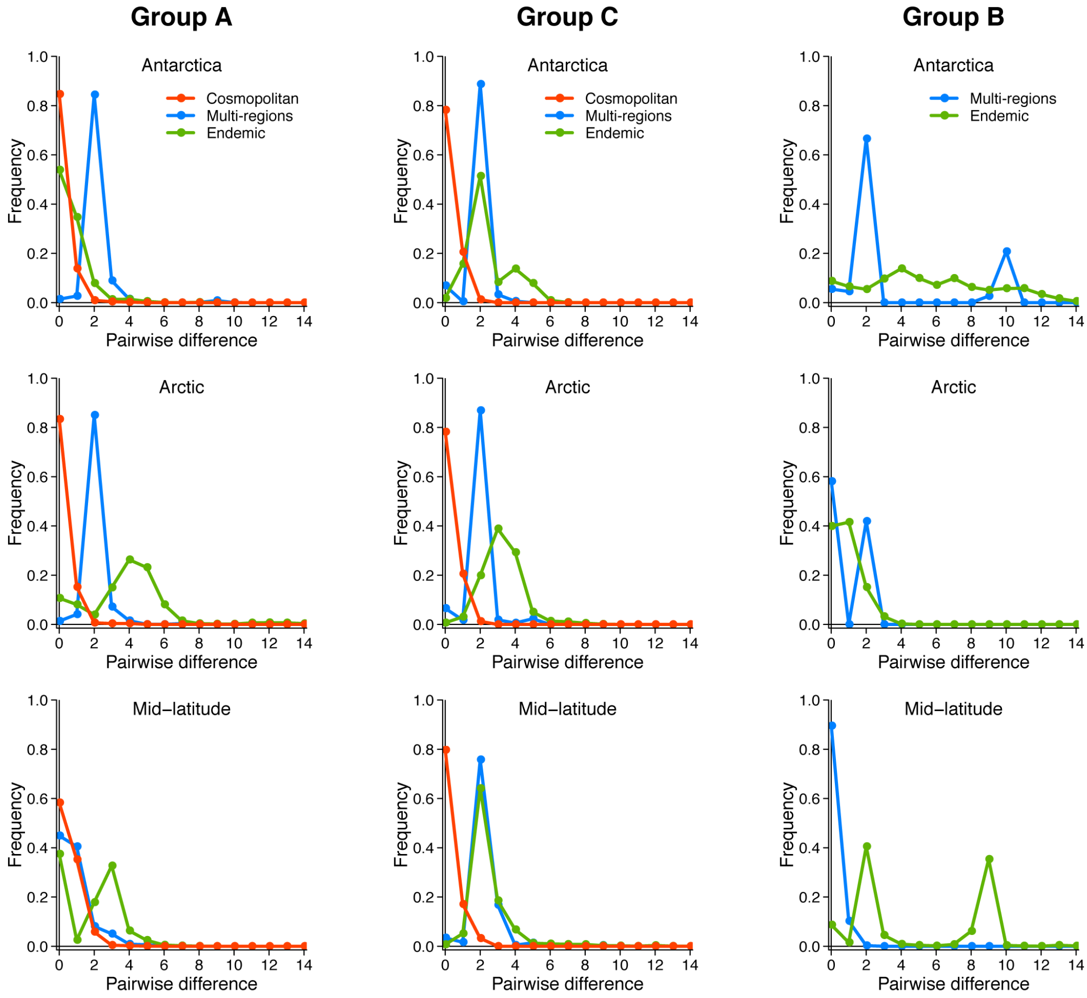

Figure S15: Mismatch distribution based on the number of pairwise differences in each *Raphidonema* Group (A–C) from each region. The lines represent the distribution of the observed number of pairwise differences of each *Raphidonema* group. Regions (Antarctica, Arctic, and mid-latitude) and distribution types (cosmopolitan, multi-regions, endemic) are distinguished. Calculations were performed for all distribution types of *Raphidonema* Groups A and C, for which various cosmopolitan phylotypes were detected and had a star-like phylogeny. On the other hand, calculations for only multi-regions and endemic phylotypes were performed for *Raphidonema* Group B, because no variation was found in cosmopolitan phylotypes.

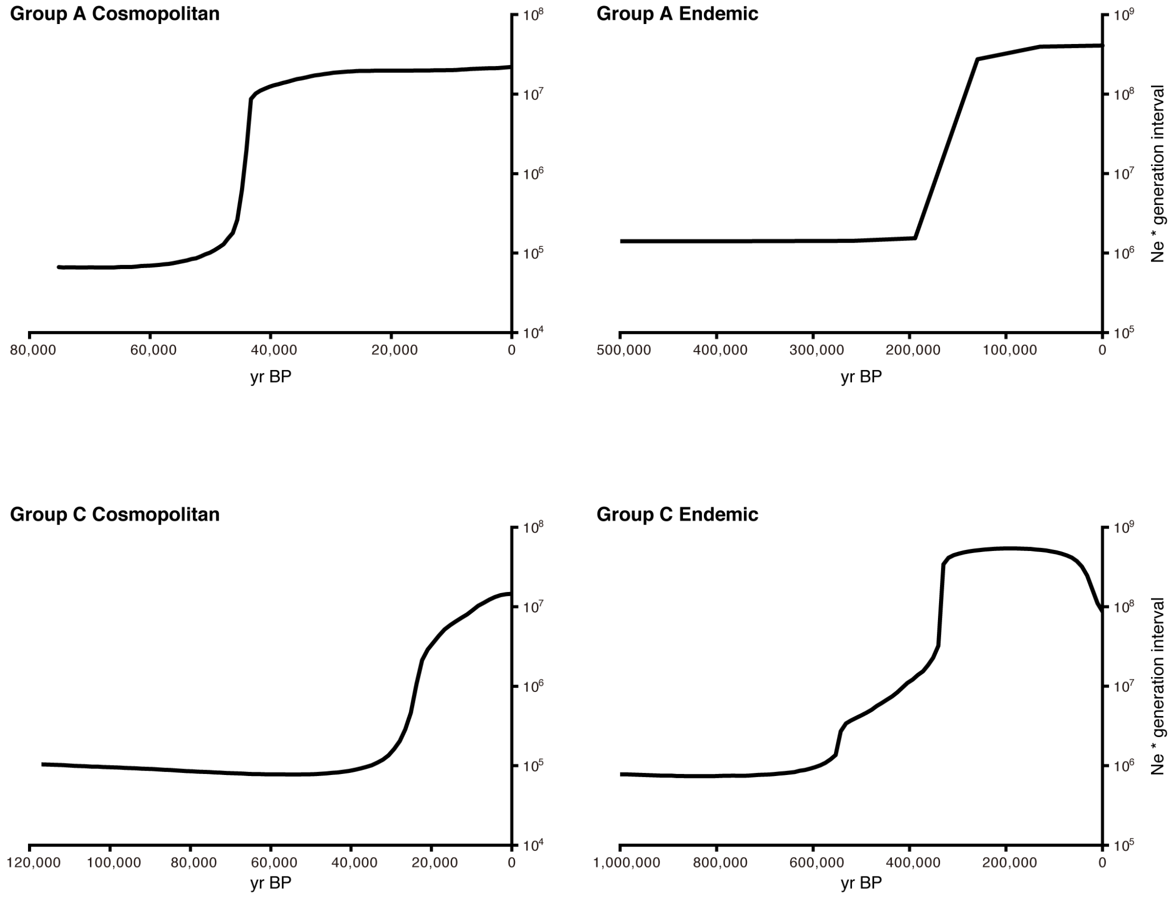

Figure S16: Bayesian skyline plots that show the Ne*t, where Ne stands for the effective population size and t stands for the generation intervals over time in each *Raphidonema* Group (A and C). Lines represent the median parameter estimates. The x axis indicates years before present; the y axis indicates the effective population size multiplied by the generation interval (Ne*t).

Table S1: List of ice core samples usedno in this study.

Ice core no.	Ice core no.	Depth (m)	Age	Successful amplification by PCR of ITS2 region
No.1	GGC2-27	3.85	AD 2001	no
No.2	GGC2-156	11.08	AD 1,984	no
No.3	GGC2-281	17.30	AD 1,967	no
No.4	GGC2-300	18.25	AD 1,965	no
No.5	GGC2-334	19.99	AD 1,963	yes
No.6	GGC2-353	20.99	AD 1,962	no
No.7	GGC2-449	25.80	AD 1,950	no
No.8	GGC2-491	27.86	AD 1,943	no
No.9	GGC2-528	29.69	AD 1,934	yes
No.10	GGC2-629	34.89	AD 1,918	yes
No.11	GGC2-992	54.05	AD 1,833	yes
No.12	GGC2-1193	63.46	AD 1,781	yes
No.13	GGC1-2202	68.60	–	yes
No.14	GGC1-2436	75.97	–	no
No.15	GGC1-2612	80.61	–	yes
No.16	GGC1-2672	81.83	7,969 – 8,017 cal yr bp	yes
No.17	GGC1-2684	82.07	–	yes
No.18	GGC1-2715	82.69	–	yes
No.19	GGC1-2854	85.52	8,016 – 8,137 cal yr bp	yes

Ages of samples No.1–12 were determined by the annual layers of pollen grains [S10]. Ages of samples No.16 and No.19 were determined by radio-carbonisotope [S11]. Ages of samples No.13–15, 17, and 18 were not determined, but appears to be close to the age of No. 16.

Table S2: List of modern snow samples used in this study.

Region	Sample location	Site	Lat	Long	Altitude (m)	Year	Site condition	Reference
Greenland	Greenland Ice sheet, Tugto Glacier	IF13	77.89473	-68.77335	1001	2012.July	red snow on the glacier	[S12]
Greenland	Greenland Ice sheet	IH2	69.56110	-49.34715	1107	2011.August	red snow on the glacier	[S12]
Svalbard	Foxfonna glacier	FX S3	78.13747	16.15794	642	2011.August	red snow on the glacier	[S12]
Svalbard	Foxfonna glacier	FX S4	78.12687	16.20279	755	2011.August	red snow on the glacier	[S12]
Svalbard	Longyearbreen glacier	LY S3	78.17579	15.49680	417	2011.August	red snow on the glacier	[S12]
Svalbard	Longyearbreen glacier	LY S4	78.16565	15.46344	580	2011.August	red snow on the glacier	[S12]
Svalbard	Austre Brøggerbreen	SP	78.89904	11.82895	130	2008.July	red snow on the glacier	[S12]
Alaska	Juneau ice field	TK1	58.64446	-134.21068	1040	2001.July	red snow on the glacier	[S12]
Alaska	Gulkana glacier	GU S5	63.28771	-145.40551	1680	2001.August	red snow on the glacier	[S12]
Alaska	Gulkana glacier	GU S4	63.28068	-145.41242	1585	2005.June	red snow on the glacier	[S12]
Alaska	Harding ice field	HD	60.15278	-149.77992	1150	2014.August	red snow on the glacier	[S12]
Antarctica	Riiser-Larsen	Riiser-Larsen	-66.78765	50.56543	500	2008.February	non-glacier based seasonal snow	[S12]
Antarctica	Yukidori Valley	Yukidori Valley	-69.23935	39.76112	50	2008.January	non-glacier based seasonal snow	[S12]
Antarctica	Johnsons Glacier, Livingston Island	CH1	-62.65595	-60.36640	5	2015.January	non-glacier based seasonal snow	[S12]
Antarctica	Johnsons Glacier, Livingston Island	CH5	-62.65588	-60.36582	5	2015.January	non-glacier based seasonal snow	[S12]
Antarctica	Johnsons Glacier, Livingston Island	CH6	-62.65563	-60.36594	5	2015.January	non-glacier based seasonal snow	[S12]
Antarctica	Johnsons Glacier, Livingston Island	CH7	-62.65604	-60.36558	5	2015.January	non-glacier based seasonal snow	[S12]
Antarctica	Glaciar Rocoso, Livingston Island	RC4	-62.71219	-60.40879	35	2015.January	non-glacier based seasonal snow	[S12]
Antarctica	Glaciar Rocoso, Livingston Island	RC5	-62.71250	-60.40926	30	2015.January	non-glacier based seasonal snow	[S12]
Antarctica	Glaciar Rocoso, Livingston Island	RC7	-62.71508	-60.41130	10	2015.January	non-glacier based seasonal snow	[S12]
Antarctica	Hurd Glacier-Sally Rocks lobe, Livingston Island	SA.1R	-62.70138	-60.41831	35	2015.January	non-glacier based seasonal snow	[S12]
Antarctica	Hurd Glacier-Sally Rocks lobe, Livingston Island	SA.2R	-62.70153	-60.41784	12	2015.January	non-glacier based seasonal snow	[S12]
Antarctica	Hurd Glacier-Sally Rocks lobe, Livingston Island	SA.3R	-62.70243	-60.41806	0	2015.January	non-glacier based seasonal snow	[S12]
Antarctica	Hurd Glacier-Sally Rocks lobe, Livingston Island	SA.4R	-62.70299	-60.41846	0	2015.January	non-glacier based seasonal snow	[S12]
Mid-latitude	No. 31 Glacier, Suntar-Khayata Mountains, Russia	SHA	62.59513	140.90641	2611	2012.July	red snow on the glacier	This study
Mid-latitude	Ürümqi Glacier No. 1, Tien Shan Mountains, China	UM S5	43.10679	86.80594	4010	2014.August	red snow on the glacier	This study
Mid-latitude	Gregoriev Glacier, Tien Shan Mountains, Kyrgyz	Kir S1	41.94847	77.91836	4250	2006.August	surface ice	This study
Mid-latitude	Gregoriev Glacier, Tien Shan Mountains, Kyrgyz	Kir S2	41.95168	77.91848	4297	2006.August	surface ice	This study
Mid-latitude	Gregoriev Glacier, Tien Shan Mountains, Kyrgyz	Kir S3	41.95818	77.91647	4367	2006.August	surface ice	This study
Mid-latitude	Gregoriev Glacier, Tien Shan Mountains, Kyrgyz	Kir S5	41.96773	77.91317	4469	2006.August	snow on the glacier	This study
Mid-latitude	Fedchenko Glacier, Pamir, Tajikistan	Fed	38.700	72.333	4040	2009.August	red snow on the glacier	This study
Mid-latitude	Tateyama Mountains, Japan	TA R2	36.58838	137.60390	2450	2011.June	non-glacier based seasonal snow	This study
Mid-latitude	Tateyama Mountains, Japan	TA Raicho	36.58838	137.60390	2450	2012.June	non-glacier based seasonal snow	This study
Mid-latitude	Tateyama Mountains, Japan	TA RA1	36.58838	137.60390	2450	2018.June	non-glacier based seasonal snow	This study

Table S3: Number of reads for each taxonomy group in the ice core and various regions.

		Ice cores	Japan	China	Tajikistan	Kyrgyz	Russia
Chlorophyceae	*Sanguina* group 1 [a]	0	0	0	0	0	2
	Sanguina group 2 [b]	0	70,908	0	211,858	94	26,287
	Chloromonadinia-snow group 1	0	63	0	0	0	0
	Chloromonadinia-snow group 2	0	0	0	0	0	13
	Chloromonadinia-snow group 3	15,463	0	14	0	88	0
	Chloromonadinia-snow group 4	16,374	7	0	0	1,527	68
	Chloromonadinia-snow group 5	20	0	0	0	0	0
	Chloromonadinia-snow group 6	0	0	0	0	0	366
	Chloromonadinia-snow group 7	0	0	0	0	0	237
	Chloromonadinia-snow group 8	4	0	0	0	0	0
	Chloromonadinia-snow group 9	0	539	0	0	0	0
	Chloromonadinia-snow group 10	7,000	0	33	0	289	389
	Chloromonadinia-snow group 11	8,102	0	38	0	0	0
	Chloromonadinia-snow group 12	0	0	0	0	0	2
	Chloromonadinia-snow group 13	0	3	0	0	0	5
	Chloromonadinia-snow group 14	3	0	0	0	0	0
	Chloromonadinia-snow group 15	116	0	0	0	13	0
	Chloromonadinia-snow group 16	486	0	0	0	0	0
	Chloromonadinia-snow group 17	468	0	0	0	0	0
	Chloromonadinia-snow group 18	0	0	0	0	0	31
	Chloromonadinia-snow group 19	0	0	0	0	0	265
	Chloromonadinia-snow group 20	0	19	0	0	12	0
	Chloromonadinia-snow group 21	0	0	0	0	0	9
	Chloromonadinia-snow group 22	0	287	21,734	0	2,454	107
	Chloromonadinia-snow group 23	0	467	0	0	0	0
	Chloromonadinia-snow group 24	0	4,790	0	0	0	0
	Chloromonadinia-snow group 25	0	0	0	0	0	4
	Chloromonadinia-snow group 26	0	0	0	0	0	361
	Chloromonadinia-snow group 27	0	0	0	0	0	600
	Moewusinia group	0	0	0	0	10	0
	Monadinia group	13,635	0	11	0	27	0
	Reinhardtinia group 1	1,158	0	0	0	516	0
	Reinhardtinia group 2	2,605	0	0	0	78	0
	Reinhardtinia group 3	2,687	0	0	0	0	0
	Stephanosphaerinia group 1	36,197	0	36	0	1,748	0
	Stephanosphaerinia group 2	3,745	0	0	0	61	0
	unnamed group	40	0	0	0	943	0
Trebouxiophyceae	*Chlorella* group 1	83,659	0	2	0	885	0
	Chlorella group 2	66,711	0	41	0	744	0
	Elliptochloris group	0	0	0	0	0	2
	Raphidonema group	425,945	0	373	0	14,666	1,614
	Trebouxia group 1	0	0	0	0	0	6
	Trebouxia group 2	12	0	18	0	0	0
	Trebouxia group 3	0	0	5	0	0	0
	Neocystis group	46	0	0	0	0	0
Ulvophyceae	*Chamaetrichon* group	77	0	0	0	0	0
	Planophila group	101	0	0	0	0	0
	Total	684,654	77,083	22,305	211,858	24,155	30,368

[a] Formerly known as *Chlamydomonas*-snow group 1

[b] Formerly known as *Chlamydomonas*-snow group 2

Table S4: Ratio for each taxonomy group in the ice core and various regions.

		Ice cores	Japan	China	Tajikistan	Kyrgyz	Russia
Chlorophyceae	*Sanguina* group 1 [a]	0	0	0	0	0	0.01
	Sanguina group 2 [b]	0	92.0	0	100	0.4	86.6
	Chloromonadinia-snow group 1	0	0.1	0	0	0	0
	Chloromonadinia-snow group 2	0	0	0	0	0	0.04
	Chloromonadinia-snow group 3	2.3	0	0.1	0	0.4	0
	Chloromonadinia-snow group 4	2.4	0.01	0	0	6.3	0.2
	Chloromonadinia-snow group 5	0.003	0	0	0	0	0
	Chloromonadinia-snow group 6	0	0	0	0	0	1.2
	Chloromonadinia-snow group 7	0	0	0	0	0	0.8
	Chloromonadinia-snow group 8	0.001	0	0	0	0	0
	Chloromonadinia-snow group 9	0	0.7	0	0	0	0
	Chloromonadinia-snow group 10	1.0	0	0.1	0	1.2	1.3
	Chloromonadinia-snow group 11	1.2	0	0.2	0	0	0
	Chloromonadinia-snow group 12	0	0	0	0	0	0.01
	Chloromonadinia-snow group 13	0	0.004	0	0	0	0.02
	Chloromonadinia-snow group 14	0.0004	0	0	0	0	0
	Chloromonadinia-snow group 15	0.02	0	0	0	0.1	0
	Chloromonadinia-snow group 16	0.1	0	0	0	0	0
	Chloromonadinia-snow group 17	0.1	0	0	0	0	0
	Chloromonadinia-snow group 18	0	0	0	0	0	0.1
	Chloromonadinia-snow group 19	0	0	0	0	0	0.9
	Chloromonadinia-snow group 20	0	0.02	0	0	0.05	0
	Chloromonadinia-snow group 21	0	0	0	0	0	0.03
	Chloromonadinia-snow group 22	0	0.4	97.4	0	10.2	0.4
	Chloromonadinia-snow group 23	0	0.6	0	0	0	0
	Chloromonadinia-snow group 24	0	6.2	0	0	0	0
	Chloromonadinia-snow group 25	0	0	0	0	0	0.01
	Chloromonadinia-snow group 26	0	0	0	0	0	1.2
	Chloromonadinia-snow group 27	0	0	0	0	0	2.0
	Moewusinia group	0	0	0	0	0.04	0
	Monadinia group	2.0	0	0.05	0	0.1	0
	Reinhardtinia group 1	0.2	0	0	0	2.1	0
	Reinhardtinia group 2	0.4	0	0	0	0.3	0
	Reinhardtinia group 3	0.4	0	0	0	0	0
	Stephanosphaerinia group 1	5.3	0	0.2	0	7.2	0
	Stephanosphaerinia group 2	0.5	0	0	0	0.3	0
	unnamed group	0.01	0	0	0	3.9	0
Trebouxiophyceae	*Chlorella* group 1	12.2	0	0.01	0	3.7	0
	Chlorella group 2	9.7	0	0.2	0	3.1	0
	Elliptochloris group	0	0	0	0	0	0.01
	Raphidonema group	62.2	0	1.7	0	60.7	5.3
	Trebouxia group 1	0	0	0	0	0	0.02
	Trebouxia group 2	0.002	0	0.1	0	0	0
	Trebouxia group 3	0	0	0.02	0	0	0
	Neocystis group	0.01	0	0	0	0	0
Ulvophyceae	*Chamaetrichon* group	0.01	0	0	0	0	0
	Planophila group	0.01	0	0	0	0	0
	Total	100	100	100	100	100	100

[a] Formerly known as *Chlamydomonas*-snow group 1

[b] Formerly known as *Chlamydomonas*-snow group 2

Table S5: Number of reads and unique sequences in total and in the *Raphidonema* group.

Samping site	name	Sequencing reads in total	Sequencing reads in *Raphidonema* group	Unique sequences in total	Unique sequences in *Raphidonema* group
Suntar-Khayata glacier	SHA	30,368	1,614	1,022	74
Raicho sawa	TA R2	25,122	0	1,005	0
Raicho sawa	TA Raicho	16,208	0	1,367	0
Raicho sawa	TA RA1	35,753	0	916	0
Ürümqi Glacier No. 1	UM S5	22,305	373	740	35
Fedchenko Glacier	Fed	211,858	0	3,968	0
Gregoriev Glacier	Kir S1	2,036	803	251	134
Gregoriev Glacier	Kir S2	10,135	7,662	1,057	863
Gregoriev Glacier	Kir S3	10,560	5,717	1,202	752
Gregoriev Glacier	Kir S5	1,424	484	151	84
Ice core on Gregoriev Glacier	No. 5	195,365	176,398	4,516	3,795
Ice core on Gregoriev Glacier	No. 9	8,580	7,317	690	585
Ice core on Gregoriev Glacier	No. 10	14,985	3,489	1,264	457
Ice core on Gregoriev Glacier	No. 11	6,970	1,271	848	237
Ice core on Gregoriev Glacier	No. 12	18,576	17,034	1,398	1,281
Ice core on Gregoriev Glacier	No. 13	2,681	128	117	20
Ice core on Gregoriev Glacier	No. 15	81,373	69,332	4,281	3,601
Ice core on Gregoriev Glacier	No. 16	131,255	9,951	5,632	1,020
Ice core on Gregoriev Glacier	No. 17	59,254	7,222	4,283	1,083
Ice core on Gregoriev Glacier	No. 18	43,281	16,361	3,192	1,242
Ice core on Gregoriev Glacier	No. 19	122,334	117,442	4,101	3,841
Total		1,050,423	442,598	25,039	8209

Table S6: Number of reads, unique sequences, and 98% OTU analysis of ITS2 sequences in the *Raphidonema* group in the various regions.

	Mid-latitude	Ice cores	Antarctica	Arctic	Total
Number of reads	16,653	425,945	139,972	311,079	893,649
98% OTU number	33	95	64	82	222
Unique sequence numbers	1,489	8,072	5,721	10,350	22,389

Table S7: PERMANOVA analysis of ITS2 sequences based on unique sequences in the *Raphidonema* group.

	Antarctica	Arctic	Mid-latitude	Ice cores
Antarctica				
Arctic	0.0002*			
Mid-latitude	0.0001*	0.0004*		
Ice cores	0.0001*	0.0001*	0.0088*	

* Statistically significant after Bonferroni's correction at $P < 0.01$.

Table S8: Classification of distribution type for this study.

<table>
<tr><th>Type</th><th>Distribution type</th><th>Detecting region</th></tr>
<tr><td>Cosmopolitan</td><td>Both poles and mid-latitude</td><td>Antarctica – Svalbard – Greenland – Alaska – Mid-latitude</td></tr>
<tr><td rowspan="3">Multi-regions</td><td>Antarctica and mid-latitude</td><td>Antarctica – Mid-latitude</td></tr>
<tr><td>Arctic and mid-latitude</td><td>Svalbard – Greenland – Alaska – Mid-latitude</td></tr>
<tr><td>Both poles only</td><td>Antarctica – Svalbard – Greenland – Alaska</td></tr>
<tr><td rowspan="2">Endemic</td><td>Arctic only</td><td>Svalbard – Greenland – Alaska</td></tr>
<tr><td>One region</td><td>Antarctica
Greenland
Svalbard
Alaska
Mid-latitude</td></tr>
</table>

Table S9: Numbers and ratio for each distribution type in the *Raphidonema* group based on unique sequences.

Distribution type	Unique sequences numbers				Unique sequences (%)			
	Antarctica	Arctic	Mid-latitude	Ice cores	Antarctica	Arctic	Mid-latitude	Ice cores
Both poles and mid-latitude	323	323	21	319	5.6	3.1	1.4	4.0
Antarctica and mid-latitude	34	-	2	34	0.6	-	0.1	0.4
Arctic and mid-latitude	-	246	35	242	-	2.4	2.4	3.0
Both poles only	1,568	1,568	-	-	27.4	15.1	-	-
Antarctica only	3,796	-	-	-	66.4	-	-	-
Arctic only	-	8,213	-	-	-	79.4	-	-
Mid-latitude	-	-	1,431	1,302	-	-	96.1	16.1
Ice cores only	-	-	-	6,175	-	-	-	76.5
Total	5,721	10,350	1,489	8,072	100.0	100.0	100.0	100.0

Table S10: Numbers and ratio for each distribution type in the *Raphidonema* group based on read sequences.

Distribution type	Sequencing reads				Sequencing reads (%)			
	Antarctica	Arctic	Mid-latitude	Ice cores	Antarctica	Arctic	Mid-latitude	Ice cores
Both poles and mid-latitude	27,963	169,893	466	56,147	20.0	54.6	2.8	13.2
Antarctica and mid-latitude	142	-	46	1,468	0.1	-	0.3	0.3
Arctic and mid-latitude	-	3,410	3,387	10,220	-	1.1	20.3	2.4
Both poles only	7,760	69,132	-	-	5.5	22.2	-	-
Antarctica only	104,107	-	-	-	74.4	-	-	-
Arctic only	-	68,644	-	-	-	22.1	-	-
Mid-latitude	-	-	12,754	261,060	-	-	76.6	61.3
Ice cores only	-	-	-	97,050	-	-	-	22.8
Total	139,972	311,079	16,653	425,945	100.0	100.0	100.0	100.0

We found that only limited unique sequences are distributed across all of the regions (mid-latitudes, 1.4%; Antarctica, 5.6%; Arctic, 3.1%). They account for a large proportion of the sequencing reads in the polar regions, but for a small proportion in the mid-latitudes (mid-latitudes, 2.8%; Antarctica, 20.0%; Arctic, 54.6%). These results suggested that a limited number of snow algae in the *Raphidonema* group detected from mid-latitude regions was globally dispersed across the two poles. The mid-latitude samples analyzed in this study were located in the Northern Hemisphere, which may have resulted in a larger amount of the both poles and mid-latitude phylotypes being detected in the Arctic as compared with the Antarctic.

Table S11: Number of unique sequences for each phylogenetic group of *Raphidonema* and each distribution type.

	Group A	Group B	Group C	Group D	Group E	Others
Both poles and mid-latitude	205	1	116	0	0	1
Antarctica and mid-latitude	13	18	3	0	0	0
Arctic and mid-latitude	123	0	117	2	1	3
Both poles only	1,115	2	439	0	10	2
Antarctica only	1,321	2,263	179	1	30	2
Arctic only	3,279	38	3,178	11	1,433	274
Mid-latitude	255	13	28	7	1,073	55
Ice cores only	429	83	1,242	119	4117	185
Total reads	6,740	2,418	5,302	140	6,664	522

Table S12: Number of total sequences for each phylogenetic group of *Raphidonema* and each distribution type.

Distribution type	Group A	Group B	Group C	Group D	Group E	Others	Total
Both poles and mid-latitude	133,446	2,798	118,211	0	0	14	254,469
Antarctica and mid-latitude	212	1,343	101	0	0	0	1,656
Arctic and mid-latitude	13,218	0	3,553	190	21	35	17,017
Both poles only	42,459	15	28,103	0	6,296	19	76,892
Antarctica only	55,737	47,247	783	2	333	5	104,107
Arctic only	24,903	517	18,165	50	20,798	4,211	68,644
Mid-latitude	1,533	38	699	635	266,665	4,244	273,814
Ice cores only	5,109	796	19,879	1,070	68,553	1,643	97,050
Total sequence reads	276,617	52,754	189,494	1,947	362,666	10,171	893,649

Table S13: Maximum likelihood estimates of demographic parameters of the phylotypes based on the coalescent model.

Group A

Phylotypes	τ	θ_0	θ_1	Log-Likelihood
Cosmopolitans	33.8 – 33.9	0.108 – 0.010	0.217 ± 0.002	-39,824
Multi-regions	11.0	$(2.19 \pm 1.64) \times 10^{-5}$	2.35 ± 0.02	-85,267
Endemics	22.0	1.27×10^{-5}	3.35 ± 0.02	-216,873

Group C

Phylotypes	τ	θ_0	θ_1	Log-Likelihood
Cosmopolitans	7.74 ± 0.64	0.0179 ± 0.4723	0.698 ± 0.004	-76,979
Multi-regions	3.10 ± 0.03	$(0.417 \pm 2.82) \times 10^{-3}$	280 ± 11	-19,891
Endemics	0.48 ± 0.03	2.27 ± 0.03	133 ± 3	-45,236

Group B

Phylotypes	τ	θ_0	θ_1	Log-Likelihood
All	15.0	6.48 ± 0.04	6.76 ± 0.04	-196,070

Table S14: Demographic parameters taking account of the mutation rates and generation intervals based on the coalescent model.

Group A

Phylotypes	*t*	N_0	N_1	*tMRCA*$_{ML}$	*tMRCA*$_{BEAST}$
Cosmopolitans	1.4×10^7	$(0.63–6.8)\times10^5$	1.4×10^6	1.8×10^5	1.2×10^5
	$[3.6\times10^6–4.0\times10^7]$	$[3.5\times10^4–1.3\times10^6]$	$[7.6\times10^5–2.6\times10^6]$	$[4.6\times10^4–5.2\times10^5]$	$[1.5\times10^4–3.3\times10^5]$
Endemics	9.2×10^6	80	2.1×10^7	2.8×10^6	2.7×10^6
	$[2.3\times10^6–2.6\times10^7]$	[44–154]	$[1.2\times10^7–4\times10^7]$	$[7.1\times10^5–8.0\times10^6]$	$[4.4\times10^5–6.4\times10^6]$

Group C

Phylotypes	*t*	N_0	N_1	*tMRCA*$_{ML}$	*tMRCA*$_{BEAST}$
Cosmopolitans	3.2×10^6	5.6×10^5	2.2×10^6	5.8×10^5	2.1×10^5
	$[8.2\times10^5–9.2\times10^6]$	$[3.1\times10^5–1.1\times10^6]$	$[1.2\times10^4–4.2\times10^4]$	$[1.5\times10^5–1.7\times10^6]$	$[1.5\times10^4–6.2\times10^5]$
Endemics	1.9×10^5	7.2×10^6	4.2×10^8	2.1×10^6	1.5×10^6
	$[5.1\times10^4–5.7\times10^5]$	$[4.0\times10^6–1.4\times10^7]$	$[2.3\times10^8–8.0\times10^8]$	$[5.3\times10^5–6.0\times10^6]$	$[2.7\times10^5–3.7\times10^6]$

Group B

Phylotypes	*t*	N_0	N_1	*tMRCA*$_{ML}$	*tMRCA*$_{BEAST}$
All	6.2×10^6	2.0×10^7	2.1×10^7	5.6×10^6	3.1×10^6
	$[1.6\times10^6–1.8\times10^7]$	$[1.1\times10^7–3.9\times10^7]$	$[1.2\times10^7–4.1\times10^7]$	$[1.4\times10^6–1.6\times10^7]$	$[4.3\times10^5–7.9\times10^6]$

t: The population expansion time (in years ago).
N_0 : Effective population sizes before the population expansion.
N_1: Effective population sizes after the population expansion.
tMRCA$_{ML}$: Time of the most recent common ancestor (in years ago) by the ML estimation.
tMRCA$_{BEAST}$: Time of the most recent common ancestor (in years ago) by the Bayesian Inference using the BEAST.
Numbers in the braket indicate the ranges of the estimates taking account of the uncertainties of the mutation rates and the generation intervals (for ML) or 95% HPD (for the Bayesian Inference).

Supplementary References

S1. Ness RW, Morgan AD, Colegrave N, Keightley PD. Estimate of the Spontaneous Mutation Rate in *Chlamydomonas reinhardtii*. Genetics. 2012;192(4):1447-54.

S2. Williamson CJ, Cameron KA, Cook JM, Zarsky JD, Stibal M, Edwards A. Glacier Algae: A Dark Past and a Darker Future. Front Microbiol. 2019;10(524).

S3. Onuma Y, Takeuchi N, Tanaka S, Nagatsuka N, Niwano M, Aoki T. Observations and modelling of algal growth on a snowpack in north-western Greenland. The Cryosphere. 2018;12(6):2147-58.

S4. Zachos J, Pagani M, Sloan L, Thomas E, Billups K. Trends, Rhythms, and Aberrations in Global Climate 65 Ma to Present. Science. 2001;292(5517):686-93.

S5. Drummond AJ, Rambaut A, Shapiro B, Pybus OG. Bayesian Coalescent Inference of Past Population Dynamics from Molecular Sequences. Mol Biol Evol. 2005;22(5):1185-92.

S6. Suchard MA, Lemey P, Baele G, Ayres DL, Drummond AJ, Rambaut A. Bayesian phylogenetic and phylodynamic data integration using BEAST 1.10. Virus Evolution. 2018;4(1).

S7. Yakimovich KM, Gauthier NPG, Engstrom CB, Leya T, Quarmby LM. A Molecular Analysis of Microalgae from Around the Globe to Revise Raphidonema (Trebouxiophyceae, Chlorophyta). J Phycol. 2021;57(5):1419-32.

S8. Mai U, Sayyari E, Mirarab S. Minimum variance rooting of phylogenetic trees and implications for species tree reconstruction. PLoS One. 2017;12(8):e0182238.

S9. Tria FDK, Landan G, Dagan T. Phylogenetic rooting using minimal ancestor deviation. Nature Ecology & Evolution. 2017;1(7):0193.

S10. Takeuchi N, Sera S, Fujita K, Aizen VB, Kubota J. Annual layer counting using pollen grains of the Grigoriev ice core from the Tien Shan Mountains, central Asia. Arct Antarct Alp Res. 2019;51:299-312.

S11. Takeuchi N, Fujita K, Aizen VB, Narama C, Yokoyama Y, Okamoto S, et al. The disappearance of glaciers in the Tien Shan Mountains in Central Asia at the end of Pleistocene. Quat Sci Rev. 2014;103:26-33.

S12. Segawa T, Matsuzaki R, Takeuchi N, Akiyoshi A, Navarro F, Sugiyama S, et al. Bipolar dispersal of red-snow algae. Nature Communications. 2018;9(1):3094.
